# LL-37: Structures, Antimicrobial Activity, and Influence on Amyloid-Related Diseases

**DOI:** 10.3390/biom14030320

**Published:** 2024-03-08

**Authors:** Surajit Bhattacharjya, Zhizhuo Zhang, Ayyalusamy Ramamoorthy

**Affiliations:** 1 School of Biological Sciences, Nanyang Technological University, 60 Nanyang Drive, Singapore 637551, Singapore; 2 Department of Chemistry, Biomedical Engineering, Macromolecular Science and Engineering, Michigan Neuroscience Institute, The University of Michigan, Ann Arbor, MI 48109, USA; zhizhuoz@umich.edu; 3 National High Magnetic Field Laboratory, Department of Chemical and Biomedical Engineering, Florida State University, Tallahassee, FL 32310, USA

**Keywords:** antimicrobial peptides, host defense peptides, LL37, structure, biophysical, human antimicrobial peptides

## Abstract

Antimicrobial peptides (AMPs), as well as host defense peptides (HDPs), constitute the first line of defense as part of the innate immune system. Humans are known to express antimicrobial precursor proteins, which are further processed to generate AMPs, including several types of α/β defensins, histatins, and cathelicidin-derived AMPs like LL37. The broad-spectrum activity of AMPs is crucial to defend against infections caused by pathogenic bacteria, viruses, fungi, and parasites. The emergence of multi-drug resistant pathogenic bacteria is of global concern for public health. The prospects of targeting antibiotic-resistant strains of bacteria with AMPs are of high significance for developing new generations of antimicrobial agents. The 37-residue long LL37, the only cathelicidin family of AMP in humans, has been the major focus for the past few decades of research. The host defense activity of LL37 is likely underscored by its expression throughout the body, spanning from the epithelial cells of various organs—testis, skin, respiratory tract, and gastrointestinal tract—to immune cells. Remarkably, apart from canonical direct killing of pathogenic organisms, LL37 exerts several other host defense activities, including inflammatory response modulation, chemo-attraction, and wound healing and closure at the infected sites. In addition, LL37 and its derived peptides are bestowed with anti-cancer and anti-amyloidogenic properties. In this review article, we aim to develop integrative, mechanistic insight into LL37 and its derived peptides, based on the known biophysical, structural, and functional studies in recent years. We believe that this review will pave the way for future research on the structures, biochemical and biophysical properties, and design of novel LL37-based molecules.

## 1. Introduction

Since the discovery of penicillin, antibiotics have saved millions of lives from infectious diseases. Antibiotics are still considered “magic bullets” and continue to serve as eminent drugs to reduce mortality from bacterial infections. However, as we note from the current affairs of antibiotics, these magic bullets are becoming less effective or sometimes even ineffective in curing patients in hospitals and in intensive care facilities [1,2,3]. At present, antibiotic resistance, or antimicrobial resistance (AMR), is increasing at a rapid rate across the globe, revealing serious consequences to human and animal health [4,5,6]. Notably, drug-resistant bacteria are responsible for most of the infections and deaths caused by AMR (*vide infra*). The Centers for Disease Control and Prevention (CDC), USA, published their first AMR threat report in 2013 that estimated that over 2 million people were infected by antibiotic-resistant bacteria, causing 23,000 deaths in the USA alone. In a more recent report, the CDC indicated that there are over 2.8 million antimicrobial-resistant infections and 35,000 human deaths every year [7]. In the year 2014, the government of the UK and the Welcome Trust jointly commissioned a review exercise to analyze the global economic impacts arising from AMR [8]. The landmark report of O’Neill made several vital recommendations to the international governments to tackle global AMR challenges [8]. The report also suggested that AMR could cause over 10 million deaths each year by 2050. A recent comprehensive report from the Antimicrobial Resistance Collaborators analyzed the worldwide occurrence of bacterial AMR for the year 2019 [9]. The study estimated a staggering number of deaths, 4.95 million, associated with bacterial AMR in that year. Notably, approximately 3.75 million mortalities associated with bacterial AMR were caused by the six bacterial pathogens, *Escherichia coli*, *Staphylococcus aureus*, *Klebsiella pneumoniae*, *Streptococcus pneumoniae*, *Acinetobacter baumannii*, and *Pseudomonas aeruginosa*. It is noteworthy that these bacteria are also included in the WHO-listed drug-resistant group of pathogens, ESKAPE (*Enterococcus faecium*, *Staphylococcus aureus*, *Klebsiella pneumoniae*, *Acinetobacter baumannii*, *Pseudomonas aeruginosa*, and *Enterobacter species*) [10].

To distinguish their pattern of susceptibility, antibiotic-resistant bacteria are categorized into three classes: multi-drug resistant (MDR), extremely drug resistant (XDR), and pan-drug resistant (PDR). MDR bacteria demonstrate resistance to at least one drug in three or more antimicrobial groups. The XDR group includes pathogens that are susceptible to only one or two categories of antibiotics. PDR bacteria have acquired resistance to all classes of antibiotics [11]. The AMR data analyses delineated that most of the deaths were caused by methicillin-resistant *S. aureus* and several MDR strains of Gram-negative bacteria, such as third-generation cephalosporin-resistant isolates of *E. coli* and *K. pneumoniae*, fluoroquinolone-resistant *E. coli*, and carbapenem-resistant strains of *A. baumannii* and *K. pneumoniae* [10]. In addition to these MDR strains of bacteria, the CDC of the USA has also indicated that drug-resistant strains of *Clostridioides difficile*, *Neisseria gonorrhoeae*, vancomycin-resistant *Enterococcus* (VRE), *Pseudomonas aeruginosa*, *Mycobacterium tuberculosis*, and *Salmonella* sp. are either urgent or serious threats.

Despite the rise of resistant strains of bacteria, the launch of new antibiotics that can be effective against multi-drug resistant pathogens from the major pharmaceutical industries has been extremely limited [12,13,14]. As a matter of fact, the introduction of any antibiotic is likely to be challenged by the development of resistance by the targeted bacteria. Therefore, new antimicrobial agents must be constantly developed to mitigate the acquisition of resistance among pathogenic bacteria [12,13,14]. For around four decades, the 1940s to the 1970s, pharmaceutical industries maintained a stable discovery pipeline in supplying new antibiotics. In that golden era, antibiotics were developed that could overcome the complications caused by bacterial resistance to earlier drugs. After that and in recent years, fewer antibiotics (quinupristin-dalfopristin, linezolid, and daptomycin) became available for the treatment of infections caused by MDR Gram-positive bacteria [15,16]. By contrast, there are now limited treatment options available to treat infections of MDR Gram-negative pathogens. In particular, infections caused by carbapenem-resistant Gram-negative bacteria are hard to treat with any another antibiotic [17,18]. To tackle these infections, an apparently nephrotoxic peptide antibiotic polymyxin B, or colistin, has been brought back for clinical usage [19,20].

## 2. Antimicrobial Peptides (AMPs) as Potential Alternatives to Antibiotic Resistance

**Figure 1:**
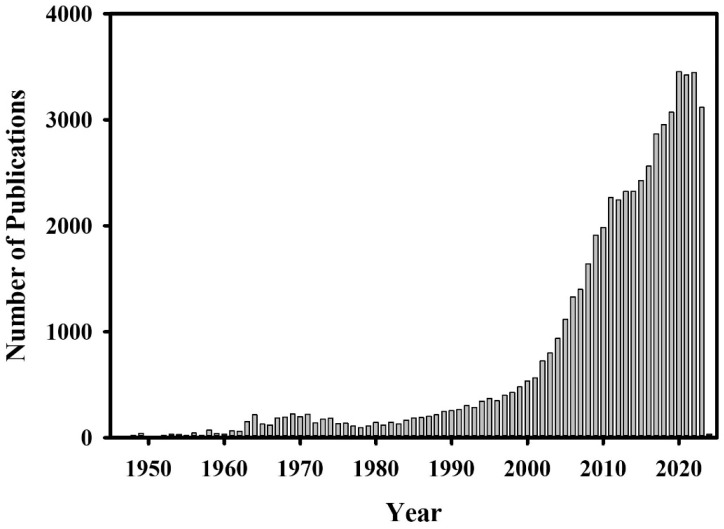
Bar diagram summarizing publications vs. years with “antimicrobial peptide” as the search option from the PubMed database.

Antimicrobial peptides (AMPs) are promising molecules of high translational potential against multi-drug resistant bacterial pathogens [21,22,23,24]. A PubMed search on “antimicrobial peptide” showed 52,404 results (Figure 1).

The increasing number of scientific publications over the years asserts that AMPs can be valuable templates for the potential development of antibiotics. Ubiquitously found in all life forms, AMPs serve as an integral component of host innate immunity in multicellular organisms, including humans [25,26,27]. Many AMPs exert a broad spectrum of activity, killing bacteria, parasites, fungi, viruses, and cancer [28,29,30,31]. As a mode of action, amphipathic AMPs lyse bacterial cells by disrupting membranes following distinct mechanisms, e.g., barrel stave, toroidal pore, or carpet [32,33,34]; the non-membrane targeting mechanisms include the inhibition of cell division, protease activity, and biosynthesis of proteins and nucleic acids. Cationic AMPs preferentially interact with negatively charged bacterial cell membranes over zwitterionic or neutral membranes of host cell membranes [25,26,27]. Gram-negative bacteria are intrinsically more resistant to several frontline antibiotics that are extremely effective in killing Gram-positive bacteria [34,35]. The lipopolysaccharide (LPS) outer membrane (or LPS-OM) of Gram-negative bacteria serves as a permeability barrier that limits intra-cellular access of several antibiotics [35,36,37,38]. Interactions of cationic AMPs with anionic phosphates of LPS or lipid A can cause an efficient permeabilization of the LPS-OM barrier [39,40,41,42]. AMP-mediated disruption of the LPS-OM is pivotal in killing Gram-negative bacteria [39,40,41,42]. The ability of AMPs in killing drug-resistant bacteria, both Gram-positive and Gram-negative, has generated a strong interest in the development of antibiotics with novel modes of action [43,44,45,46]. More recent studies have demonstrated efficacy of several AMPs against MDR-resistant strains of Gram-negative bacteria in infected animal models with low host toxicity [47,48,49,50]. Notably, large-scale genomic data analyses have revealed that bacteria are less likely to develop resistance against AMPs compared to the conventional antibiotics [51,52]. These attributes of AMPs need to be exploited for the development of anti-infective agents to treat infections of the drug-resistant bacterial pathogens. Many studies have reported the antimicrobial activities of AMPs derived from amphibians, insects, mammals, microorganisms such as fungi and bacteria, and de novo design.

In humans, tissue-specific expressions of antimicrobial proteins and peptides constitute the innate immunity to eliminate invading pathogens [53,54,55]. Based on the Antimicrobial Peptide Database (APD), there are 153 host defense peptides in humans [56]. The well-characterized human AMPs include the defensins families, α and β, cathelicidin LL37, histatins, and dermcidin. In addition, a number of human proteins, e.g., multiple types of RNases, lysozyme, chemokines, and psoriasin, exhibit antimicrobial activities. Finally, proteolytic fragments of certain native proteins are bestowed with host defense activity [57,58,59]. Table 1 shows a selected list of AMPs identified in humans.

## 3. Cathelicidin-Derived AMPs

Mainly found in higher organisms, including vertebrates and mammals, cathelicidin AMPs exert a broad spectrum of activity within the innate and adaptive host defense systems [80,81]. The cathelicidin family of AMPs are typically recognized by the presence of a conserved “cathelin” domain, ~14 KDa, in their precursor proteins [80,81]. The cathelin domain was first identified from analyses of proteolytic digestion of peptide fragments from pig leukocytes and was determined to be an inhibitor of the cysteine proteinase cathepsin L [82]. Structurally, the cathelin domain belongs to the cystatin superfamily of protease inhibitors, including cystatin (cysteine proteinase inhibitor), kininogen, and stefin proteins [82,83,84,85]. The cathelicidin protein is expressed as a precursor protein or a pre-protein that contains an N-terminal signal sequence followed by the cathelin domain and the C-terminus antimicrobial region [82,83,84,85]. The pre-protein undergoes multiple steps of proteolytic processing before cathelicidin AMP can be functionally activated [80,81]. The signal peptide is cleaved off, giving rise to the “holo-protein” during the translocation to an extra-cellular space or in zymogen granules. Further processing of the holo-protein to a pro-protein entails stabilization of the cathelin domain by the formation of two disulfide bonds. At the final stage of processing, the holo-protein is proteolytically cleaved, releasing the active forms of the antimicrobial region and the cathelin domain [80,81]. Although the cathelin domain is well conserved, the AMPs derived from cathelicidin proteins demonstrate great diversity in their amino acid sequence structure and activity [82,83,84,85]. The secondary structures of cathelicidin AMPs encompass amphipathic α-helix stabilized in the lipid membrane, disulfide bonded β-sheets, and AMPs rich in specific amino acid types [86,87,88,89,90]. In general, cathelicidin AMPs exhibit a broad spectrum of antimicrobial activity, although toxicity to cells and tissues in animal models has been observed [86,87,88,89,90]. Chicken cathelicidin AMPs or fowlicidins are extremely hemolytic although highly potent in killing wide-ranging pathogenic bacteria, including drug-resistant strains [91,92]. Table 2 summarizes a list of representative cathelicidin AMPs and their amino acid sequences, secondary structures, and activity profiles [86,87,89,93,94,95,96,97,98,99,100,101,102,103,104,105,106,107].

LL37 is the only cathelicidin-derived AMP in humans [86,108]. The 37-residue LL37 is linear in its amino acid sequence, without any disulfide bond, and helical in its structure [109,110,111]. These characteristics are widely different from disulfide-bonded β-sheet human defensin AMPs [112,113,114]. Towards the discovery of LL37, two independent studies were aimed to identify the cathelicidin gene(s) in humans using cDNA probes obtained from the homologous genes of pigs and rabbits [60,61]. Analyses of cDNA probes of pigs reported the existence of a human gene that may code for a putative 39-residue long peptide (or FALL39) as a part of a cathelin-like precursor protein [60]. The chemically synthesized FALL39 peptide demonstrated helical conformations and inhibited growth of bacterial strains of *E. coli* D21 and *B. megaterium* [60]. On the other hand, a cDNA probe based on the rabbit CAP18 gene has led to the characterization of the human CAP18 gene [61]. Western blot experiments have demonstrated the expression of CAP18 or 18 KDa precursor protein in granulocytes [61]. The 37-residue synthetic peptide of the C-terminus of CAP18 demonstrated high-affinity LPS binding and protected mice from LPS-induced endotoxic shock [62]. Another study isolated the LL37 precursor protein from human neutrophils and obtained its c-DNA clone from human myeloid cells [115]. Furthermore, analyses of total genomic DNA revealed the existence of only one cathelicidin gene in humans [115]. The 37-residue mature form of AMP of hCAP18 was isolated from granulocytes and was termed LL37, based on its first two Leu residues [115].

## 4. Importance of Structures of AMPs

AMPs are pivotal sources of natural arsenals that can be utilized to combat MDR infections [43,44,45,46]. Thus, the rational development of potent and selective antimicrobials from AMPs would require in-depth structure–activity relationship (SAR) studies. Traditionally, based on amphipathicity, AMPs are categorized as α-helix, β-sheet/β-hairpin, and non-random (no typical secondary structures). However, atomic-resolution structures of AMPs in a complex with bacterial targets are essential to generate SAR for novel antibiotics. Notably, three-dimensional structures of several AMPs are known to vary significantly when determined in cell membranes or membrane-mimicking environments [32,41,42]. In this regard, the atomic-resolution structures of several potent AMPs as a complex with an LPS outer membrane could be correlated with Gram-negative specific activity [116,117,118,119,120,121,122,123]. Cathelicidin-derived AMPs are found to be structurally diverse (Table 2). Atomic-resolution structures of several members of AMPs in the cathelicidin family have been determined in membranes or in membrane mimics (Table 2). NMR-derived structures of α-helical cathelicidin AMPs include LL37 [109,110,111], mice [124], pig [125], sheep [126], bovine [127], and fowlicidins [128,129,130]. The helical AMPs appear to be unstructured in a free solution and assume largely monomeric helical conformations in the solutions of membrane environments, e.g., detergent micelles, bicelles, nanodiscs, vesicles, or helix-promoting organic solvents. Interestingly, an oligomeric structure of fowlicidin-1, chicken cathelicidin, was determined in a solution of zwitterionic DPC detergent micelles [130]. Although the in-vivo concentrations for all AMPs are very low, the local population density is very high, enough to cause damage to the cell membrane. The lipids of the membrane have been shown to assist the self-assembly of the peptides to form an aggregate/oligomer, which is more potent in lysing bacterial cells. The oligomeric structure of fowlicidin-1 indicates membrane pore formation and cytotoxicity. The oligomerization and structures of protegrin-1, β-sheet cathelicidin from porcine, in membranes demonstrated mechanistic insights, cell selective activity, and SAR-based designs of analogs [131].

## 5. Biological Properties of LL37

LL-37, the only human cathelicidin-derived antimicrobial peptide, has long been a popular research subject because of its special abilities and vast applications. In the past 15 years, hundreds of papers have been published with LL-37 being their primary focus. Although minimal progress has been made on certain areas related to LL-37, such as the correlation between its high-resolution structure and activity, many multidisciplinary studies have shown LL-37 to be one of the most promising AMPs with a variety of applications. LL37’s functional properties are summarized below.

### 5.1. Antimicrobial and Antiviral Activities of LL-37

LL-37, though it has been proven to be useful in many ways, is, in essence, an antimicrobial peptide that is primarily used by the body to fight microorganisms like bacteria and fungi. On top of that, the antiviral ability of LL-37 has also long been a popular topic. In the past 15 years, LL-37 has been considered as a promising candidate for the treatment of a number of diseases, with the majority of them being bacterial and some being viral. Table 3 summarizes some of the diseases that have been studied with LL-37. In the majority of the cases, treatments using LL-37 were found to have a positive effect, while in others, resistance to LL-37 was reported. Apart from the specific diseases, LL-37 has also been studied extensively with certain bacteria, especially the ones under the genera Burkholderia, Neisseria, Pseudomonas, Staphylococcus, and Streptococcus. The potential possibilities to treat diseases caused by microorganisms without leading to resistance make LL-37 a promising replacement for conventional antibiotics, which has been demonstrated in some cases. However, the resistance to LL37 noticed in some diseases points to the need for further studies before progressing to the next step towards drug development.

Starting in 2008, a particular aspect of the antimicrobial activity of LL-37 has been investigated, which is its ability to inhibit the formation of bacterial biofilms [185]. A biofilm is an aggregate of bacterial cells that is covered by an extracellular polymeric substance (EPS) matrix. By forming biofilms, bacterial cells are able to protect themselves from harmful substances, such as attacks from the immune system and antibiotics. Like other antimicrobial agents, LL-37 is also prevented by bacterial biofilm from attacking the bacterial cells, which is why some bacteria exhibit resistance against LL-37. However, Overhage et al. noted that LL-37 is able to prevent the formation of biofilms through a series of mechanisms that have not yet been well understood [186]. Such mechanisms include biofilm gene suppression, bacteria adhesion inhibition, biofilm matrix degradation, bacteria cells elimination, and several other major or minor functions [185]. On the other hand, bacterial biofilms also have a variety of mechanisms that mediate the interference from LL-37, explaining why LL-37 has not yet been the solution to overcome biofilm-related challenges. That being said, certain ways to improve the antibiofilm ability of LL-37 have been proposed, such as using its synergy with other antimicrobial agents, indicating a possible therapeutic application in the future. Studies have also reported LL-37 degradation by the metalloprotease aureolysin, produced by *S. aureus* strains, suggesting the resistance of this pathogen correlating with the loss of LL-37’s antibacterial activity. On the other hand, the fragment LL-17-37 produced due to the glutamyl endopeptidase V8 protease, exhibited antibacterial activity against *S. aureus* [187,188]. There are other studies that reported the inactivation of LL-37 [156,189,190,191].

### 5.2. Anticancer Activity of LL-37

Antimicrobial peptides have also been shown to exhibit anticancer activities [28,30,31,192,193,194,195]. Since cancer cells are anionic, the cationic AMPs exhibit selectivity in targeting cancer cells in a similar manner to their selective targeting of bacterial cells. While there is significant interest in designing anticancer peptides using AMPs, the LL-37 peptide has received special attention, as it is the only cathelicidin-derived human peptide. Although the chemotactic potential of LL-37 was noticed almost immediately upon its discovery, it was only beginning around 2005 that the anticancer potential became a noteworthy aspect of this AMP [196]. In recent years, more and more research has become oriented towards the influence of LL-37 on cancer, along with the rise of research interest in cancer in the biology field in general [197,198,199]. LL-37 has been found to have contrasting effects on different types of cancers: for certain cancers, such as breast, lung and ovary cancer, LL-37 is tumorigenic and facilitates the cancer formation process, while in other cancers, like colon and gastric cancer, LL-37 has been proven to be anticancer. Verjans et al. suggest that this result may be explained by the difference in receptors that respond to LL-37 in different cells [200]. Even though LL-37 is tumorigenic in some cases, it can still be used to help treat such cancer by acting as a biomarker [197]. In ovarian cancer, LL-37 has been found to be over-expressed, and it is able to facilitate cancer spread in many ways, like inducing cell proliferation and cell invasion. Similar results were found for lung, breast, and pancreas cancer and malignant melanoma, while the tumorigenic effect of LL-37 can also be extrapolated for prostate cancer and skin squamous cell carcinoma. In all these types of cancers, treatment of recombinant LL-37 has shown a positive correlation with tumor development. On the other hand, the over-expression of LL-37 is also observed in colon cancer, but it was also found in this case that LL-37 can lead to a decrease in cancer tissues. For gastric cancer, hematologic malignancy, and oral squamous cell carcinoma, a lower expression of LL-37 was found, and it has also been proven to down-regulate cancer development, showing an anticancer effect. More studies are needed to fully understand the mechanism behind LL-37’s involvement in cancer growth, but current results do suggest some possible therapeutic applications of LL-37 in cancer treatments.

### 5.3. Other Functional Properties of LL-37

Another noteworthy aspect of LL-37 is its role in the human immune system [201,202,203]. LL-37 has been shown to be able to attract immune cells to fight microbial infection. The first group of cells attracted is the neutrophils, which form the first line of defense against infection. These cells can also produce more LL-37, leading to a positive feedback loop. Recent research has also noted that in the case of serum amyloid A inflammation and sepsis, LL-37 performs immunoregulatory functions by inhibiting neutrophil migration, which is another novel aspect of the immune activity of LL-37. In addition to neutrophils, LL-37 is also able to modulate monocytes, macrophages, and dendritic cells. Monocytes, sometimes referred to as adult stem cells, are able to differentiate into macrophages and dendritic cells, which are important components of the immune system that fights off infection. A crucial role of LL-37 in modulating the differentiation process, as well as regulating the immunological functions of macrophages and dendritic cells, has also been proven. Further immunoregulatory functions of LL-37 on lymphocytes, mast cells, and MSCs have also been noted, though minimal discoveries have been made. Another important function of LL-37 in the immune system is its ability to neutralize lipopolysaccharides (LPSs), which can be crucial in bacterial infections.

The wound healing and angiogenesis ability of LL-37 has also been recognized for a long time [204]. This aspect of LL-37 may also act as a contributing factor in the curing of microbial diseases and cancer. Recently, Chinipardaz et al. also discovered a potentially important role of LL-37 in bone and periodontal regeneration [165]. This, combined with the wound healing ability of LL-37, may point to a potential application in treating oral cavity diseases. Furthermore, connections of LL-37 with amyloid proteins have also been reported in recent studies. Certain connections between LL-37 and beta-amyloid, which is a possible cause of Alzheimer’s disease, have been proven, and the hypothesis that LL-37 may be involved in the pathogenesis of Alzheimer’s disease has been proposed, with a need for further examination [205,206]. Similar connections between LL-37 and IAPP, which is linked with type 2 diabetes, have also been found, and follow-up studies in this area are also needed [207]. Overall, the vast function of LL-37 opens it up to a variety of therapeutic applications in many different fields, while an increasing number of studies are forthcoming.

## 6. Structures of LL-37

Ever since its discovery, LL-37 has been studied not only in its original monomeric form but also in more complex structures obtained under different conditions. Studies have found that when treated with detergents under certain conditions, LL-37 can form monomers as well as oligomerize into dimers and tetramers [111,208,209]. Furthermore, derivatives of LL-37, such as the core peptide (LL-3717-29) and KR-12 (LL-3717-29), have also been studied extensively [210,211]. These structures, each with unique features, can become useful for research purposes to better understand the different functional properties of LL-37 and its derivatives and also for further development towards pharmaceutical applications.

### 6.1. Monomeric Structures of LL-37

LL-37 has been shown to undergo a structural transition from an unstructured monomer in solution to a helical structure in any of the following conditions: (i) at high peptide concentrations, (ii) in the presence of salt, and (iii) in the presence of detergents or lipids [212]. Atomic-resolution three-dimensional structures of the LL-37 monomer have been reported under different environments with different detergents. A solution NMR study reported a helix-break-helix conformation for LL-37 reconstituted into dodecylphosphocholine (DPC) micelles [109]. This study also found that the unstructured N- and C-termini are solvent exposed, while the structured C-terminal helix is protected from the solvent, and the N-terminal helical domain is more dynamic. The peptide is bound to the surface of DPC micelles with the hydrophobic I13, F17, and I20 residues and a salt bridge between E16 and K12 stabilizing the break between the two helices.

**Figure 2:**
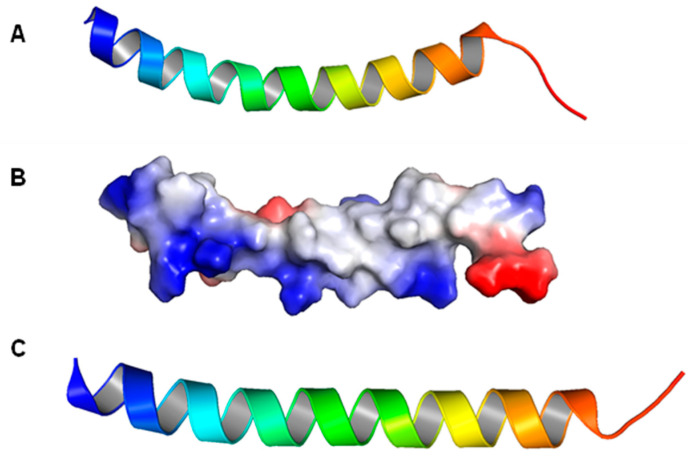
Structures of LL-37 monomers. (**A**) Structure of LL-37 monomer determined in deuterated SDS micelles (2K6O). (**B**) Electrostatic potential of LL-37 monomer in deuterated SDS micelles; blue represents positively charged residues, red represents negatively charged residues, and white represents hydrophobic residues. (**C**) Structure of LL-37 monomer determined in DPC micelles (5NMN).

Wang et al. reported a standard LL-37 monomer structure (PDB number 2K6O), obtained using a three-dimensional triple-resonance NMR technique [111]. The conditions used were 303 K and pH 5.4, and deuterated SDS (sodium dodecyl sulfate) detergent micelles were used. The structure that they determined, as shown below, is a curved alpha helix with a well-defined helical region covering residues 2–31, while the residues at the C-terminus appear to be disordered (Figure 2A). The structure also contains a notable bent located between residues 14–16, which is consistent with the helix-break-helix structure predicted in other publications. In addition, the LL-37 helix appears to be amphipathic, with about half of the residues, namely residues L2, F5, F6, I13, F17, I20, V21, I24, F27, L28, and L31, being hydrophobic and located on the concave side (Figure 2B). The hydrophilic residues are located on the other side, with the exception of residue S9, which is on the hydrophobic side and divides that region into two parts. The author also proposed that the helix-break-helix structure may be a result of the hydrophobic packing between residues I13 and F17, which are located next to each other with a bend in between.

In another study, an LL-37 structure determined from a different detergent, dioctanoylphosphatidylglycerol (D8PG) micelles, was reported, using the same technique and experimental conditions as described above. The obtained structure appears to be similar, if not identical, to the above-mentioned LL-37 structure determined for SDS micelles. However, because D8PG has the same head structure as many anionic phosphatidylglycerols, the author also used it to investigate the interaction between LL-37 and anionic PGs. Direct evidence for interactions between the aromatic rings of the phenylalanine residues as well as the arginine residues of LL-37 and the PGs was found. Sancho-Vaello et al. reported a monomeric LL-37 structure (5NMN), obtained with DPC micelles using X-ray crystallography, which also has similar features to the other structures determined from detergent micelles (Figure 2C) [208]. This structure is less bent compared to the structure determined in SDS micelles, with residues 35–37 missing on the model, possibly because they are disordered and cannot be detected by the X-ray diffraction technique.

### 6.2. Oligomeric Structures of LL-37

**Figure 3:**
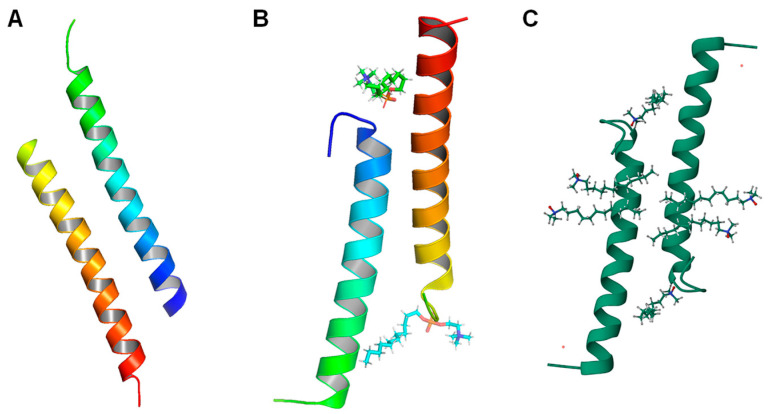
Structures of LL-37 dimers. (**A**) LL-37 dimer structure in detergent-free environment (5NNM). (**B**) LL-37 dimer structure in DPC micelles (5NNT). (**C**) LL-37 dimer structure in LDAO micelles (5NNK).

In addition to the structure of the LL-37 monomer, the structures of the oligomers of LL-37 are important to better understand the stability of the peptide against enzymatic degradation. LL-37 has been shown to form aggregates at high peptide concentrations in solution [212,213,214,215]. Sancho-Vaello et al. also explored the structure of LL-37 dimers in a detergent-free environment (5NNM), as well as in DPC (5NNT) and LDAO (5NNK) micelles [208]. When there is no detergent present, the dimeric LL-37 appears to be an antiparallel dimer made from two alpha helices without supercoiling (Figure 3A). The two monomers are similar to the monomer obtained in DPC (5NMN), especially since there is very little bending compared to the SDS and D8PG ones. Each helix in this dimer extends to around 5 nm, with approximately two turns shifted at each terminus, leading to a 3.5 nm interface. The hydrophilic interactions that link the two dimers are formed by the residues S9, K12, and E16 of the two monomers, whereas intermolecular stabilization is mainly provided by the H-bond and four salt bridges. In addition, the hydrophobic residues at the interface form a hydrophobic core in the dimer that extends to the C-terminus, which also contributes to the high stability of the dimer. The authors also noted a discontinuity in the hydrophobic region, which is the positively charged residue K10. The opposite side of the dimer is dominated by the hydrophilic residues, with 20 of those being positively charged and eight being negatively charged, leading to a +12 overall charge. Another point worth noting is that like the DPC monomer described above, eight of the 74 residues are not present in the structure, indicating a disordered region at the C-terminus. The same applies for the two other dimers made in detergents.

The dimer structures obtained in DPC and LDAO micelles are highly similar antiparallel dimers, but they differ strongly from the one obtained in a detergent-free environment (Figure 3B,C). The authors found that only the core region of the two dimers can align with the detergent-free dimer as a result of the structural remodeling caused by detergents. Specifically, the remodeling at the N-terminus shortens each monomer to about 4 nm and the interface to 2.5 nm. The residues L1 to R7 at the N-terminus, unlike in the dimer obtained without detergents, appear to be randomly coiling. This remodeling allows residues F5 and F6 to be exposed so that they can form hydrophobic contacts with the alkyl chains of the detergents, which is assisted by residues I24 and F27 of the second monomer. This conformation is further stabilized by the H bond between residue K10 and residues G3 and F5. Further conformational changes at residues L1, I13, and I17 can also be attributed to the influence of the detergent. The residues at the C-terminus also experience a shift in conformation, though not as significant as the remodeling at the N-terminus. Because of the change in structure, the bond that connects the two monomers in this case is formed by residue S9 on one monomer and residue E16 on the other. The authors also found that these dimer structures can also form tetramers and other fiber-like oligomers with a head-to-tail arrangement. The oligomers are primarily stabilized by residues F5, F6, and F27, which form hydrophobic scaffolds to embed detergent molecules. The exact structure of the tetramer (7PDC) is documented in another paper written by the same group of authors.

**Figure 4:**
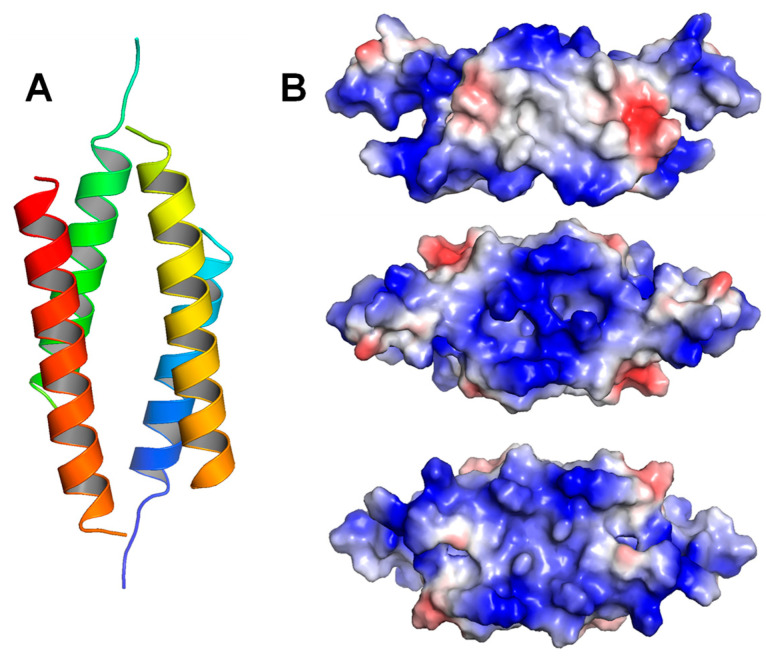
The structures of LL-37 tetramer. (**A**) LL-37 tetramer structure in DPC micelles (7PDC). (**B**) The three interfaces of the tetramer, with the first one being hydrophobic and the other two being polar.

The LL-37 tetramer structure was also obtained with DPC micelles and modeled using a crystallization technique (7PDC) [209]. The tetramer is made by two asymmetric dimers, each containing two antiparallel monomers (Figure 4A). This structure is a narrow tetrameric channel with a 4 nm length, and its monomers are similar to those in the DPC dimers (5NNT) and the DPC monomer (5NMN). Disordered residues are observed at both termini, leaving a well-defined helical region between residues 6 and 30. However, the dimer structure seen in this tetramer is very different from the dimers described above, and the new structure seems to provide a better structural fit. The tetramer appears to be asymmetrical, but the structure does form a continuous and positively charged inner cavity. As a result of this asymmetric structure, there exist three interfaces, with one being hydrophobic and the others being charged and polar (Figure 4B). These interfaces are stabilized by salt bridges and hydrophilic contacts. The authors also suggested that the influence of the three interfaces might be the cause of this unique conformation, as opposed to being caused by detergents like the dimers described above. In the center of the tetramer, there is a chlorine ion trapped by two R23 residues and coordinated by two water molecules. The core itself is stabilized by many hydrogen bond interactions as well as 15 water molecules that are also found within the channel, while no water molecules are present in the surrounding of the tetramer. The authors also noted two aromatic grindles on the tetramer, each formed by two F17 and two F27 residues, which indicate the membrane integration potential of this structure. With follow-up tests, the presence of this tetramer in membrane-like environments is confirmed in the paper, as well as the conductivity of the channel to pass molecules into cells.

## 7. LL-37 Derivatives

### 7.1. Core Peptide and Related Fragments

**Figure 5:**
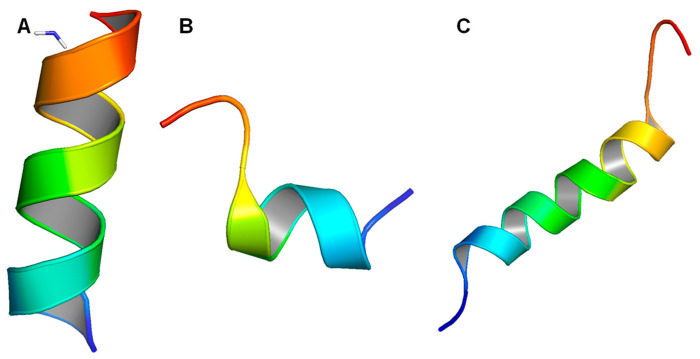
The structures of LL-37 core peptide and other fragments. (**A**) LL-37 core peptide structure in D8PG and deuterated SDS (2FBS). (**B**) LL-37 N-terminal fragment structure in D8PG and deuterated SDS (2FBU). (**C**) LL-37 C-terminal fragment structure in D8PG and deuterated SDS (2FCG).

Because LL-37 has been studied extensively in the past two decades, its original structure and the structures of its many derivatives have been explored in great detail. One of the first derivatives that draws a lot of attention is its core peptide, LL-3717-29. The core peptide is 13 residues long, and it is referred to in such a way because it was thought at the time to be the smallest fragment that exhibits AMP properties [210]. Li et al. studied the structure of the LL-37 core peptide with solution NMR under a 298 K temperature 5.4 pH and using both D8PG and deuterated SDS as detergents (2FBS). The structure obtained is an amphipathic alpha helix, which appears to be the same under the two detergent environments (Figure 5A). The authors found that about half of the residues are located on the hydrophobic surface, while the other half are on the hydrophilic one. For the hydrophilic surface, it is evident that the positively charged residues dominate the region, just like in many other LL-37 structures, and this suggests that the peptide is more ideal for targeting negatively charged membranes. The authors also noted an analogical structure to the core peptide, aurein 1.2, which also has antimicrobial and anticancer properties. By studying these two peptides along with a bacterial membrane anchor, the authors proposed that hydrophobic clusters that involve aromatic rings might be crucial for membrane binding. Apart from the core peptide, the article also reported two other derivatives of LL-37, which are the N-terminal fragment (LL-37_1–12_) and the C-terminal fragment (LL-37_13–37_). The N-terminal fragment (2FBU) obtained appears to be disordered for the most part, with only a one-turn helix covering residues 3–7 present (Figure 5B). For this peptide, only 62% of the backbone angles are located in the most favored region, in contrast to the result of 100% for the core peptide. The backbone angles in the less favored region are located in the disordered region, namely residues 8–12. The authors also focused on the hydrophobic clusters that involve aromatic–aromatic interaction, just like that noted in the core peptide. It was found that a single hydrophobic cluster created by the aromatic rings on P5 and P6 as well as the side chain of L2 leads to the poor hydrophobicity of the peptide, which could be the reason for this fragment’s poor AMP and anticancer ability. In addition to that, this cluster may also play a role in the oligomerization of LL-37, as described in the last section. The C-terminal fragment (2FCG) contains a well-defined alpha-helical structure between residues 17–29, corresponding perfectly to the core peptide (). The rest of the fragment, residues 13–16 and 30–37, appears to be disordered, and their backbone angles are also located in less favored regions similar to that of the N-terminal fragment. A weaker AMP ability of this fragment compared to the core peptide and the whole peptide was also noted, which may as well be a result of the interference of this poorly defined region with membrane binding.

**Figure 6:**
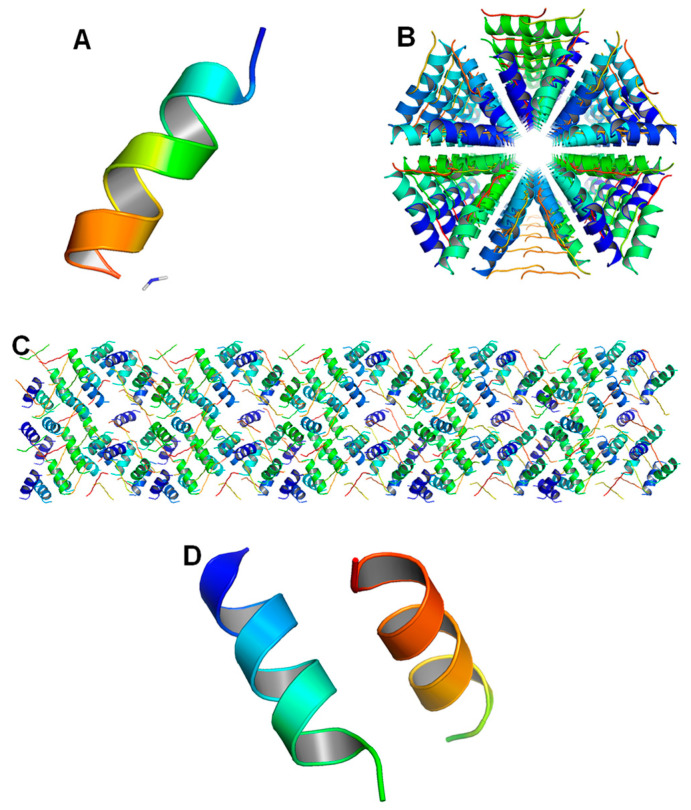
The structures of LL-37 core peptide variations. (**A**) LL-37 retro core peptide structure in D8PG and SDS (2F3A). (**B**) LL-37 core peptide fiber structure, viewed from the top (6S6M). (**C**) LL-37 core peptide fiber structure, viewed from the side (6S6M). (**D**) LL-37 core peptide I24C mutant structure in sodium acetate (7NPQ).

Li et al. also reported the structure of the retro core peptide of LL-37 (2F3A) [216], which was investigated as an analog of aurein 1.2. Obtained in the presence of SDS and D8PG, the structure appears to be alpha-helical with a well-defined helix covering residues 2–12 (Figure 6A). Similar to all LL-37 related peptides, the retro core peptide is also amphipathic with hydrophobic residues on one side and hydrophilic residues on the other. One interesting feature of this peptide is that the aromatic rings on residues F3 and F13 are located in the same chemical environment in SDS and D8PG. Because F13 penetrates the micelles deeper than F3 and the NOE pattern of F3 is similar to what is found in bacterial membrane anchors, it was concluded that F3 might also be serving the same purpose in this case. Engelberg and Landau further explored the structure of fibrils formed by LL-37 core peptides (6S6M) using crystallization techniques [7,217]. In a detergent-free environment with sodium acetate used as salt, the core peptides assemble into a densely packed hexameric fibrous structure with a central pore, composed of numerous four-helix bundles as the building unit (Figure 6B,C). These bundles, each containing a hydrophobic core that provides stabilization for the structure, are highly positively charged. The polar interactions between the bundles, especially the salt bridge formed by adjacent helices, allow the formation of the hexameric fibrils. The resulting fibrils are found to be highly stable and are capable of interacting with bacterial membranes. In another article, Engelberg et al. also reported a mutant of the core peptide, I24C (7NPQ) (Figure 6D) [8,218]. This mutant is found initially as dimers connected by a disulfide bond at the C24 residue, but they can further assemble to form fibrils using a network of interaction, particularly salt bridges, as a stabilizing factor. The fibrils contain a hydrophobic core, which extends through the structure.

### 7.2. KR-12 Based Peptides

**Figure 7:**
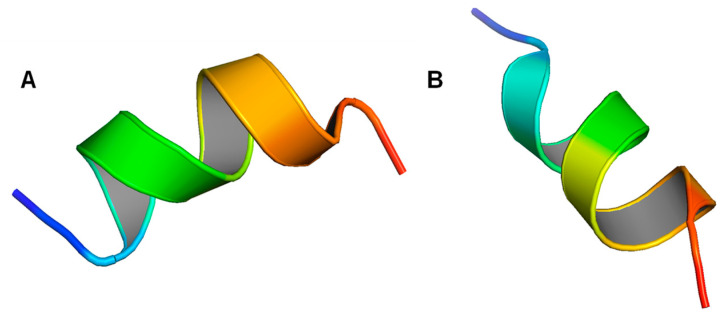
The structures of LL-37 derivative KR-12. (**A**) LL-37 KR-12 structure in lysophosphatidylglycerol and SDS (2NA3). (**B**) LL-37 retro KR-12 structure in lysophosphatidylglycerol and SDS (2NAL).

KR-12 (LL-37_18-29_) is one of the most important derivatives of LL-37 because of its outstanding AMP properties and low toxicity to human cells. Gunasekera et al. studied the structure of KR-12 (2NA3) and retro KR-12 (2NAL) using solution NMR with lysophosphatidylglycerol and SDS as the detergents [211]. KR-12 is in the form of an alpha helix, with a clear helical structure between residues 3–11 (Figure 7A). Like the other peptides, KR-12 has the charged and hydrophilic residues on one side and the hydrophobic ones on the other, while having a net positive charge. The overall structure is not much different from the core peptide, which is only one residue more than KR-12. However, it was noticed that KR-12 can form cyclic dimers that possess enhanced AMP ability, although the dimer structure was not reported on the PDB. The retro KR-12, being simply the reverse of KR-12, shows a very similar structure to the KR-12 structure (Figure 7B). The only noticeable difference between the two is the marginally decreased AMP ability seen in retro KR-12 compared to KR-12. Yun et al. also found an analog of KR-12 (6M0Y) in another article, which may have the potential to become a cosmetic product [219].

## 8. Solid-State NMR Studies on the Mechanism of Membrane Disruption by LL-37

A complete understanding of the function of an antimicrobial peptide can only be accomplished by determining the atomic-resolution three-dimensional structure, dynamics, and membrane folding/topology of the peptide in a lipid membrane environment. A detergent micelle is not a suitable membrane mimetic to study antimicrobial peptides because of the following reasons: (i) it does not have an appropriate hydrophobic membrane core to enable native folding of the hydrophobic domains (like the transmembrane domain) of the peptide, and (ii) its curvature can distort the overall shape of the amphipathic structural regions, such as by bending the helix. In addition, the absence of native-like lipid–peptide interactions both with the head groups and hydrophobic acyl chains is unlikely to allow the self-assembly of peptides and oligomer formation to occur. Therefore, it is essential to use a better membrane mimetic. A lipid bilayer is considered to be a better mimetic, and the feasibility to alter the lipid/membrane composition to mimic bacterial versus mammalian cell membrane is an added advantage. Since lipid bilayers are fluid and dynamic but an isotropic phase, they pose challenges for atomic-resolution structural studies. On the other hand, solid-state NMR techniques are well-suited to studying such dynamic systems [220,221,222,223,224,225].

**Figure 8:**
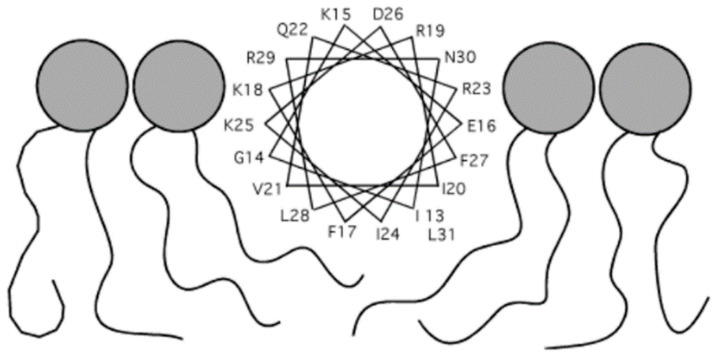
Cartoon showing the orientation of helical LL-37 peptide with respect to the lipid bilayer. As mentioned in the text, magic angle spinning (MAS) solid-state NMR of LL-37 reconstituted in a lipid bilayer, solution NMR of LL-37 in detergent micelles, and circular dichroism (CD) experiments on micelles or lipid vesicles containing LL-37 revealed the amphipathic helical structure of LL-37 [109,207] The use of static solid-state NMR experiments on mechanically aligned lipid bilayers containing ^15^N-labeled LL-37 rendered the in-plane orientation of the peptide [212]. The figure is reprinted with copyright permission from Ref. [212].

Solid-state NMR is a technique used to determine the structure and dynamics of a variety of solids and semi-solids (examples include liquid crystalline systems), and it is an ideal approach to investigate biological membranes that are difficult to study with other biophysical techniques like solution NMR or crystallization techniques [226,227,228,229,230]. In the case of LL-37, solid-state NMR experiments were used to determine the backbone conformation, dynamics, and membrane orientation in order to determine the mechanism of lipid membrane disruption by LL-37. The cell membrane disruption process by a peptide or protein has been broadly defined using three possible mechanisms: the barrel-stave, detergent-like, and toroidal-pore mechanisms. Henzler-Wildman et al. used synthetic LL-37 peptides selectively labeled with ^15^N and/or ^13^C isotopes and model membranes composed of a combination of synthetic lipids [212]. The backbone conformation of LL-37 associated with a lipid bilayer was found to be helical using ^13^C CP-MAS (cross-polarization magic angle spinning) solid-state NMR experiments, which was found to be in excellent agreement with CD experiments. Then, using static cross-polarization solid-state NMR experiments performed on mechanically aligned lipid bilayers containing site-specifically ^15^N-labeled LL-37, the helix was found to be oriented nearly parallel to the bilayer surface (or nearly perpendicular to the bilayer normal) (Figure 8). This observation ruled out the barrel-stave mechanism of membrane disruption for which the peptide should be assembled to form channel-like structures with the helical axis oriented parallel to the bilayer normal (or transmembrane topology). Then, to measure the LL-37-induced perturbation of the lipid bilayer structure, static ^31^P NMR experiments were carried out on mechanically aligned lipid bilayers and also on multilamellar vesicles. The observed ^31^P NMR spectra revealed the absence of isotropic peaks that would arise from the peptide-induced fragmentation and formation of any small “micellar-like” lipid aggregates, which ruled out a detergent-like membrane of membrane disruption. The observed aligned, anisotropic ^31^P NMR spectral line shapes were consistent with a carpet/toroidal-type mechanism in which the bilayer surface association of LL-37 disrupted the head group region of lipids. Differential scanning calorimetry (DSC) experiments revealed LL-37’s ability to induce positive curvature on the lipid bilayer, which is indicative of a toroidal pore-type mechanism. Taken together, these NMR and DSC experimental results indicated that a toroidal pore-type membrane disruption is the likely possibility. Mechanisms of membrane interaction and disruption by LL-37 have also been investigated by other approaches [86,110,214,231,232,233,234,235,236].

**Figure 9:**
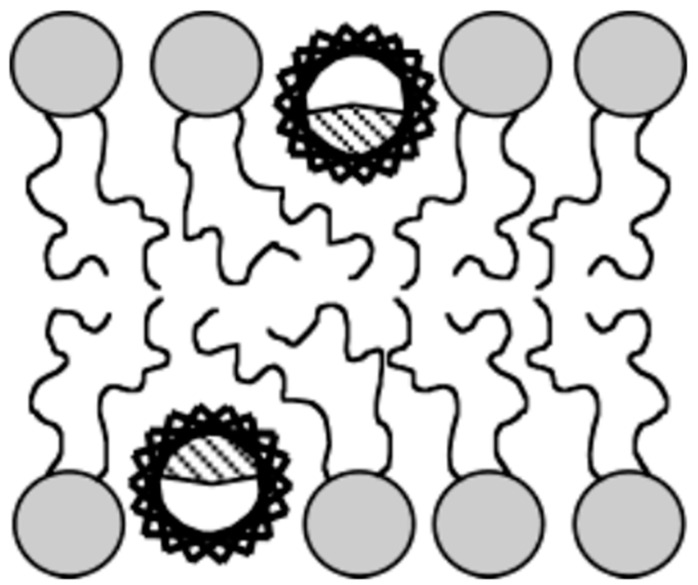
Cartoon showing the insertion of LL-37 helices into the lipid membrane bilayer with the hydrophobic region of the helix shown as shaded [237]. In addition to the solid-state NMR experiments used to determine the membrane orientation of LL-37 (see Figure 8), ^2^H solid-state NMR experiments on vesicles containing deuterated lipids and LL-37 were used to determine the peptide-induced disorder of the acyl chains of lipids, as shown [237]. The figure is reprinted with copyright permission from Ref. [237].

To investigate the mechanism by which LL-37 perturbs the hydrophobic core of the lipid bilayer, a series of static 2H solid-state NMR and DSC experiments were carried out on lipid vesicles [237]. The 2H quadrupole couplings measured from 2H-labeled lipids were used to determine an LL-37-induced disorder of the acyl chains of lipids. The peptide-induced disorder of the hydrophobic core of the lipid bilayer was found to be maximal for the lower-order carbons of the lipid acyl chains. These results along with the above-mentioned NMR findings confirmed that amphipathic helices of LL-37 associate with the lipid bilayer surface through electrostatic interactions and inserts into the hydrophobic region of the membrane stabilized via hydrophobic interactions with lipid acyl chains. These interactions act together to cause membrane disruption (Figure 9). Further evidence showed that LL-37 insertion also alters the material properties of the membrane and that the order of the bilayer influences the depth of the insertion, as well as the effectiveness of the disruption.

## 9. Influence of LL-37 on Amyloid Aggregation

**Figure 10:**
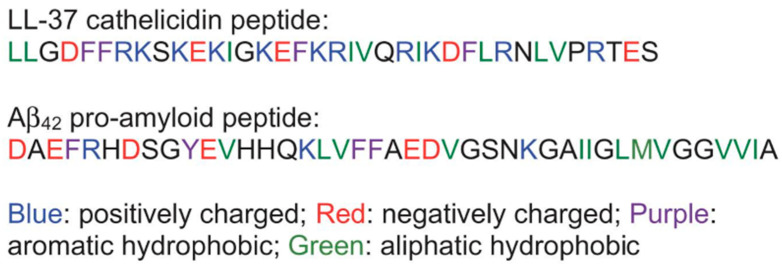
Amino acid sequences of LL-37 and Aβ_42_ with charges and aromatic and aliphatic amino acids identified.

With many properties of LL-37 being uncovered over the past decades, its interactions with amyloid β (Aβ) have also been investigated. Studies have reported the misfolding, aggregation, oligomer formation, and fibril formation of an intrinsically disordered peptide Aβ [238]. These properties of Aβ have been shown to be associated with the pathogenesis of Alzheimer’s disease (AD). Studies have reported the aggregation-induced oligomer formation and membrane-disrupting properties of Aβ peptides [239,240,241,242,243,244,245,246,247,248,249,250]. Studies have also reported the interaction between beta-amyloid and LL-37 peptides [205]. In addition, recent studies have also reported neuroinflammation and a variety of in vivo properties of LL-37 [251,252]. De Lorenzi et al. explored the possible influence of LL-37 on the amyloid aggregation of the Aβ42 isomer [205]. Through surface plasmon resonance imaging (SPRi) in vitro experiments, De Lorenzi et al. found evidence showing that LL-37 binds specifically to Aβ. Transmission electron microscopy (TEM) analysis of the aggregates showed that LL-37 inhibits Aβ42’s ability to form amyloid fibril structures, which is associated with the pathogenesis of AD. Circular dichroism (CD) spectroscopy also showed that LL-37 directly interacts with Aβ42 to prevent the formation of a β-sheet secondary structure and therefore the fibril formation. It was also found that when allowed to interact with each other, the toxicities of LL-37 and Aβ42 to neurons were both significantly reduced. Based on these findings, it is proposed that the AD pathogenesis may be associated with the expression of LL-37 and its balance with Aβ42. As De Lorenzi et al. pointed out, this finding only marks the starting point of research regarding the interaction between LL-37 and Aβ42. More investigations and evidence are needed to fully understand this relationship between the two peptides (Figure 10).

A 37-residue human hormone amylin (also called IAPP, islet amyloid polypeptide) aggregates to form amyloid fibrils in the pancreatic islet cells [253,254,255,256]. The self-assembly of IAPP results in the formation of oligomeric intermediates that are shown to exhibit major cell toxicity. Therefore, there is significant interest in the development of inhibitors of IAPP’s aggregation [257,258,259,260,261]. Remarkably, nanomolar affinity of LL-37 binding with IAPP (islet amyloid polypeptide) has been shown to effectively suppress the amyloid aggregation of IAPP and its cell toxicity [207].

## 10. Summary and Future Directions

There is considerable interest and an urgent need for the development of novel compounds to overcome the increasing bacterial resistance. While antimicrobial peptides have been thought to be promising candidates, and significant research progress has been reported towards understanding their mechanisms of action, there are very few peptide-based compounds that have successfully become pharmaceutical compounds. On the other hand, studies have explored other types of biological activities for AMPs. For example, the only type of cathelicidin-derived AMP in humans, LL-37, has drawn much attention due to its numerous biological activities, including antimicrobial activities, LPS-neutralizing activities, and modulation of immune and inflammatory pathways [262,263,264,265,266]. While LL-37’s mechanisms of antibacterial activity have been reasonably well investigated through biophysical studies, further studies to better understand its other biological roles, such as its effects on immune system function, are essential to fully exploit its potential therapeutic applications and side effects. In addition, LL-37’s interference with other biological processes such as protein misfolding and aggregation and biocondensation is an exciting area for future research. In particular, further studies to fully understand the effects of LL-37 on the molecular processes underlying amyloid aggregation, membrane disruption, oligomer formation, and neuronal cell toxicity associated with the pathology of Alzheimer’s disease would be useful.

## Figures and Tables

**Table 1:** A representative list of human antimicrobial peptides (AMPs) with antibacterial activity.

Name	Sequence	Net Change	Activity^@^	Ref.
LL37	LLGDFFRKSKEKIGKEFKRIVQRIKDFLRNLVPRTES	+6 (pI 10.6)	G+/G−	[60,61,62]
α-Defensin HNP-1	ACYCRIPACIAGERRYGTCIYQGRLWAFCC	+3 (pI 8.68)	G+/G−	[63]
α-Defensin HNP-2	CYCRIPACIAGERRYGTCIYQGRLWAFCC	+3 (pI 8.67)	G+/G−	[63]
α-Defensin HNP-3	DCYCRIPACIAGERRYGTCIYQGRLWAFCC	+2 (pI 8.33)	G+/G−	[63]
α-Defensin HNP-4	VCSCRLVFCRRTELRVGNCLIGGVSFTYCCTRV	+4 (pI 8.98)	G+/G−	[64]
α-Defensin HD-5	ATCYCRTGRCATRESLSGVCEISGRLYRLCCR	+4 (pI 8.96)	G+/G−	[65]
Histatin 3	DSHAKRHHGYKRKFHEKHHSHRGYRSNYLYDN	+5 (pI 9.9)	G+/G−	[66]
β-Defensin HBD-1	DHYNCVSSGGQCLYSACPIF TKIQGTCYRGKAKCCK	+4 (pI 8.87)	G+/G−	[67]
β-Defensin HBD-2	GIGDPVTCLKSGAICHPVFCP RRYKQIGTCGLPGTKCCKKP	+6 (pI 9.3)	G+/G−	[68]
β-Defensin HBD-3	GIINTLQKYYCRVRGGRCAVLSCLPKEEQ IGKCSTRGRKCCRRKK	+11 (pI 10)	G+/G−	[69]
β-Defensin HBD-4	FELDRICGYGTARCRKKCRSQEYRIGRCPNTYACCLRKWDESLLNRTKP	+7 (pI 9.45)	G+/G−	[70]
Dermcidin	SSLLEKGLDGAKKAVGGLGKLGKDAVEDLESVGKGAVHDVKDVLDSV	−2 (pI 5.07)	G+/G−	[71]
Granulysin	GRDYRTCLTIVQKLKKMVDKPTQRSVSNAATRVCRTGRSRWRDVCRNFMRRYQSRVTQGLVAGETAQQICEDLR	+11 (pI10.83)	G+/G−	[72]
Ubiquicidin	KVHGSLARAGKVRGQTPKVAKQEKKKKKTGRAKRRMQYNRRFVNVVPTFGKKKGPNANS	+19 (pI12.15)	G+/G−	[73]
Thrombocidin-1	AELRCMCIKTTSGIHPKNIQSLEVIGKGTHCNQVEVIATLKDGRKICLDPDAPRIKKIVQKKLAGDES	+4 (pI 9.05)	G+/G−	[74]
Hepcidin 25 (LEAP-1)	DTHFPICIFCCGCCHRSKCGMCCKT	+2 (pI 8.22)	G+/G−	[75]
Neuropeptide α-MSH	SYSMEHFRWGKPV	+1 (pI 8.33)	G+	[76]
PACAP Neuropeptide	HSDGIFTDSYSRYRKQMAVKKYLAAVLGKRYKQRVKNK	+9 (pI 10.41)	G+/G−	[77]
KDAMP	RAIGGGLSSVGGGSSTIKY	+2 (pI 9.99)	G−	[78]
DEFB114	DRCTKRYGRCKRDCLESEKQIDICSLPRKICCTEKLYEEDDMF	0 (pI 6.37)	G+/G−	[79]

^@^G+ and G− represent Gram-positive and Gram-negative bacteria, respectively.

**Table 2:** A representative list of cathelicidin-derived AMPs across several species and structural classes.

Source	Name	Sequence	Net Charge	Sec. Structure	Antibacterial Activity^@^	Toxicity	References
Human	LL37	^1^LLGDFFRKSKEKIGKEFKRIVQRIKDFLRNLVPRTES^37^	+6	Helix (NMR)	G+/G–	Hemolytic	[86]
Rhesus Monkey	RL37	^1^RLGNFFRKVKEKIGGGLKKVGQKIKDFLGNLVPRTAS^37^	+8	Helix (CD)	G+/G−	Hemolytic	[93]
Rabbit	CAP18	^1^GLRKRLRKFRNKIKEKLKKIGQKIQGFVPKLAPRTDY^37^	+12	Helix (CD)	G+/G−	Non- Hemolytic	[87]
Mice	CRAMP	^1^GLLRKGGEKIGEKLKKIGQKIKNFFQKLVPQPEQ^34^	+6	Helix (NMR)	G+/G−	Hemolytic	[94]
Guinea Pig	CAP11	^1^GLRKKFRKTRKRIQKLGRKIGKTGRKVWKAWREYGQIPYPCRI^43^-dimer -disulfide-linked	+16	ND	G+/G−	Hemolytic	[95]
Pig	Tritrpticin	^1^VRRFPWWWPFLRR^13^	+4	b-strand (NMR)	G+/G−	Hemolytic	[96]
Pig	Protegrin-1	^1^RGGRLCYCRRRFCVCVGR^18^	+7	b-sheet (NMR)	G+/G−	Hemolytic, cytotoxic	[97]
Pig	PMAP37	^1^GLLSRLRDFLSDRGRRLGEKIERIGQKIKDLSEFFQS^37^	+4	Helix (CD)	G+/G−	Hemolytic	[98]
Pig	PR39	^1^RRRPRPPYLPRPRPPPFFPPRLPPRIPPGFPPRFPPRFP^39^	+11	ND	G+/G−	ND	[99]
Bovine	Bactenecin	^1^RLCRIVVIRVCR^12^	+4	b-turn^2^	G+/G−	Non- Hemolytic	[100]
Cattle	Indolicidin	^1^ILPWKWPWWPWRR^13^	+4	b-strand (NMR)	G+/G−	Non- Hemolytic	[89]
Sheep	SMAP29	^1^RGLRRLGRKIAHGVKKYGPTVLRIIRIAG^29^	+10	Helix (NMR)	G+/G	Hemolytic	[101]
Bovine	BMAP27	^1^GRFKRFRKKFKKLFKKLSPVIPLLHLG^27^	+10	Helix (NMR)	G+/G−	Non Hemolytic	[102]
Bovine	BMAP28	^1^GGLRSLGRKILRAWKKYGPIIVPIIRIG^28^	+7	Helix (NMR)	G+/G−	Hemolytic	[102]
Bovine	BMAP34	^1^GLFRRLRDSIRRGQQKILEKARRIGERIKDIFRG^34^	+8	Helix (CD)	G+/G−	Non Hemolytic	[103]
Pig	PMAP23	^1^RIIDLLWRVRRPQKPKFVTVWVR^23^	+6	Helix (NMR)	G+/G−	Non Hemolytic	[98]
Pig	PMAP36	^1^VGRFRRLRKKTRKRLKKIGKVLKWIPPIVGSIPLGCG^37^	+13	ND	G+/G−	Hemolytic	[98]
Sheep	SMAP34	^1^GLFGRLRDSLQRGGQKILEKAERIWCKIKDIFR^33^	+5	ND	G+/G−	Hemolytic	[104]
Equine	e-CATH1	^1^KRFGRLAKSFLRMRILLPRRKILLAS^26^	+9	Helix (CD)	G+/G−	Non Hemolytic	[105]
Chicken	Fowlicidin-1	^1^RVKRVWPLVIRTVIAGYNLYRAIKKK^26^	+8	Helix (NMR)	G+/G−	Hemolytic	[91]
Chicken	Fowlicidin-2	^1^RFGRFLRKIRRFRPKVTITIQGSARFG^27^	+9	Helix (NMR)	G+/G−	Hemolytic	[91]
Chicken	Fowlicidin-3	^1^RVKRFWPLVPVAINTVAAGINLYKAIRRK^29^	+7	Helix (NMR)	G+/G−	Hemolytic	[91]
Hagfish	HFIAP-1	^1^GFFKKAWRKVKHAGRRVLDTAKGVGRHYVNNWLNRYR^37^	+10	ND	G+/G−	ND	[106]
Hagfish	HFIAP-3	^1^GWFKKAWRKVKNAGRRVLKGVGIHYGVGLI^30^	+8	ND	G+/G−	ND	[106]
Crocodile	As-CATH7	^1^KRVNWRKVGRNTALGASYVLSFLG^24^	+6	Helix (CD)	G+/G−	ND	[107]
Crocodile	As-CATH8	^1^KRVNWAKVGRTALKLLPYIFG^21^	+6	Helix (CD)	G+/G−	ND	[107]
Crocodile	Gg-CATH5	^1^TRRKWWKKVLNGAIKIAPYILD^22^	+6	Helix (CD)	G+/G−	ND	[107]
Crocodile	Gg-CATH7	^1^KRVNWRKVGLGASYVMSWLG^20^	+5	Helix (CD)	G+/G−	ND	[107]

^@^G+ and G− represent Gram-positive and Gram-negative bacteria, respectively.

**Table 3:** Bacterial and viral diseases that have been studied with LL-37 in the past 15 years.

Disease Studied	General Conclusion	Ref.
Bacterial pneumonia	Possible candidate for treatment	[132,133,134,135,136,137,138,139]
COPD	Candidate for treatment, though it may also play a role in the pathogenesis process	[140,141,142,143,144]
Infected segmental bone defects	Possible candidate for treatment	[145]
Influenza A	Possible candidate for treatment	[146,147,148,149,150,151]
Gonorrhea	Possible candidate for treatment	[152,153]
Keratitis	Possible candidate for treatment	[154,155]
Leptospirosis	Bacteria inhibits LL-37	[156]
Lupus	Possible candidate for treatment	[157,158,159,160]
Meningitis	Candidate for treatment, though resistance to LL-37 has been reported	[161,162,163]
Periodontitis	Possible candidate for treatment	[164,165,166,167,168]
Psoriasis	LL-37 plays a role in the pathogenesis process but may still be used for therapeutic purposes.	[158,160,169,170,171,172,173,174,175,176,177,178,179,180,181,182,183,184,185,186,187,188,189]
Rheumatoid arthritis	LL-37 plays a role in the pathogenesis process but may still be used for therapeutic purposes.	[158,172,173,174,175]
Sepsis	Candidate for treatment, though significant possible side effects have been noted	[176,177,178,179]
Tuberculosis	Possible candidate for treatment	[134,180,181,182,183]
Ulcerative colitis	Possible candidate for treatment	[184]

## References

[1] Taubes G. (2008). The Bacteria Fight Back. Science.

[2] Kupferschmidt K. (2016). Resistance Fighters. Science.

[3] Ikuta K.S., Swetschinski L.R., Robles Aguilar G., Sharara F., Mestrovic T., Gray A.P., Davis Weaver N., Wool E.E., Han C., Gershberg Hayoon A. (2022). Global Mortality Associated with 33 Bacterial Pathogens in 2019: A Systematic Analysis for the Global Burden of Disease Study 2019. Lancet.

[4] Morrison L., Zembower T.R. (2020). Antimicrobial Resistance. Gastrointest. Endosc. Clin. N. Am..

[5] Hutchings M.I., Truman A.W., Wilkinson B. (2019). Antibiotics: Past, present and future. Curr. Opin. Microbiol..

[6] Wang Z., Koirala B., Hernandez Y., Zimmerman M., Park S., Perlin D.S., Brady S.F. (2022). A Naturally Inspired Antibiotic to Target Multidrug-resistant Pathogens. Nature.

[7] (2019). Antibiotic Resistance Threats in the United States.

[8] O’Neill J. (2016). Tackling Drug-Resistant Infections Globally: Final Report and Recommendations.

[9] Murray C.J.L., Ikuta K.S., Sharara F., Swetschinski L., Robles Aguilar G., Gray A., Han C., Bisignano C., Rao P., Wool E. (2022). Global Burden of Bacterial Antimicrobial Resistance in 2019: A Systematic Analysis. Lancet.

[10] De Oliveira D.M.P., Forde B.M., Kidd T.J., Harris P.N.A., Schembri M.A., Beatson S.A., Paterson D.L., Walker M.J. (2020). Antimicrobial Resistance in ESKAPE Pathogens. Clin. Microbiol. Rev..

[11] Magiorakos A.-P., Srinivasan A., Carey R.B., Carmeli Y., Falagas M.E., Giske C.G., Harbarth S., Hindler J.F., Kahlmeter G., Olsson-Liljequist B. (2012). Multidrug-resistant, Extensively Drug-resistant and Pandrug-resistant Bacteria: An International Expert Proposal for Interim Standard Definitions for Acquired Resistance. Clin. Microbiol. Infect..

[12] May M. (2014). Drug Development: Time for Teamwork. Nature.

[13] Smith P.A., Koehler M.F.T., Girgis H.S., Yan D., Chen Y., Chen Y., Crawford J.J., Durk M.R., Higuchi R.I., Kang J. (2018). Optimized Arylomycins Are a New Class of Gram-negative Antibiotics. Nature.

[14] Hegemann J.D., Birkelbach J., Walesch S., Müller R. (2023). Current developments in antibiotic discovery: Global microbial diversity as a source for evolutionary optimized anti-bacterials: Global microbial diversity as a source for evolutionary optimized anti-bacterials. EMBO Rep..

[15] Madden J., Outterson K. (2023). Trends in the Global Antibiotics Market. Nat. Rev. Drug Discov..

[16] Hashemian S.M., Farhadi T., Ganjparvar M. (2018). Linezolid: A Review of Its Properties, Function, and Use in Critical Care. Drug Des. Dev. Ther..

[17] Adams-Sapper S., Nolen S., Donzelli G.F., Lal M., Chen K., Justo Da Silva L.H., Moreira B.M., Riley L.W. (2015). Rapid Induction of High-level Carbapenem Resistance in Heteroresistant Kpc-producing Klebsiella Pneumoniae. Antimicrob. Agents Chemother..

[18] Balm M.N.D., La M.-V., Krishnan P., Jureen R., Lin R.T.P., Teo J.W.P. (2013). Emergence of Klebsiella Pneumoniae Co-producing Ndm-type and OXA-181 Carbapenemases. Clin. Microbiol. Infect..

[19] Rabanal F., Cajal Y. (2017). Recent Advances and Perspectives in the Design and Development of Polymyxins. Nat. Prod. Rep..

[20] Brown P., Dawson M.J. (2017). Development of New Polymyxin Derivatives for Multi-drug Resistant Gram-negative Infections. J. Antibiot..

[21] Lazzaro B.P., Zasloff M., Rolff J. (2020). Antimicrobial peptides: Application informed by evolution. Science.

[22] Magana M., Pushpanathan M., Santos A.L., Leanse L., Fernandez M., Ioannidis A., Giulianotti M.A., Apidianakis Y., Bradfute S., Ferguson A.L. (2020). The value of antimicrobial peptides in the age of resistance. Lancet Infect. Dis..

[23] Zasloff M. (2019). Antimicrobial Peptides of Multicellular Organisms: My Perspective. Adv. Exp. Med. Biol..

[24] Bhattacharjya S., Straus S.K. (2020). Design, Engineering and Discovery of Novel α-Helical and β-Boomerang Antimicrobial Peptides against Drug Resistant Bacteria. Int. J. Mol. Sci..

[25] Haney E.F., Straus S.K., Hancock R.E.W. (2019). Reassessing the Host Defense Peptide Landscape. Front. Chem..

[26] Boman H.G. (2003). Antibacterial peptides: Basic facts and emerging concepts. J. Intern. Med..

[27] Torres M.D.T., Sothiselvam S., Lu T.K., de la Fuente-Nunez C. (2019). Peptide Design Principles for Antimicrobial Applications. J. Mol. Biol..

[28] Hoskin D.W., Ramamoorthy A. (2008). Studies on anticancer activities of antimicrobial peptides. Biochim. Biophys. Acta.

[29] Tornesello A.L., Borrelli A., Buonaguro L., Buonaguro F.M., Tornesello M.L. (2020). Antimicrobial Peptides as Anticancer Agents: Functional Properties and Biological Activities. Molecules.

[30] Kardani K., Bolhassani A. (2021). Antimicrobial/anticancer peptides: Bioactive molecules and therapeutic agents. Immunotherapy.

[31] Madera L., Hoskin D.W. (2017). Protocols for Studying Antimicrobial Peptides (AMPs) as Anticancer Agents. Methods in Molecular Biology.

[32] Nguyen L.T., Haney E.F., Vogel H.J. (2011). The expanding scope of antimicrobial peptide structures and their modes of action. Trends Biotechnol..

[33] Shai Y. (2002). Mode of action of membrane active antimicrobial peptides. Biopolymers.

[34] Matsuzaki K. (2001). Why and how are peptide-lipid interactions utilized for self defence?. Biochem. Soc. Trans..

[35] Theuretzbacher U. (2017). Global antimicrobial resistance in Gram-negative pathogens and clinical need. Curr. Opin. Microbiol..

[36] Brown D. (2015). Antibiotic Resistance Breakers: Can Repurposed Drugs Fill the Antibiotic Discovery Void?. Nat. Rev. Drug Discov..

[37] Payne D.J., Gwynn M.N., Holmes D.J., Pompliano D.L. (2007). Drugs for Bad Bugs: Confronting the Challenges of Antibacterial Discovery. Nat. Rev. Drug Discov..

[38] Willyard C. (2017). The Drug-resistant Bacteria That Pose the Greatest Health Threats. Nature.

[39] Nikaido H. (2003). Molecular Basis of Bacterial Outer Membrane Permeability Revisited. Microbiol. Mol. Biol. Rev..

[40] Zgurskaya H.I., López C.A., Gnanakaran S. (2015). Permeability Barrier of Gram-negative Cell Envelopes and Approaches to Bypass It. ACS Infect. Dis..

[41] Bhattacharjya S. (2015). NMR Structures and Interactions of Antimicrobial Peptides with Lipopolysaccharide: Connecting Structures to Functions. Curr. Top. Med. Chem..

[42] Bhattacharjya S., Mohid S.A., Bhunia A. (2022). Atomic-resolution Structures and Mode of Action of Clinically Relevant Antimicrobial Peptides. Lancet Infect. Dis..

[43] Luther A., Urfer M., Zahn M., Müller M., Wang S.-Y., Mondal M., Vitale A., Hartmann J.-B., Sharpe T., Monte F.L. (2019). Chimeric Peptidomimetic Antibiotics Against Gram-negative Bacteria. Nature.

[44] Nicolas I., Bordeau V., Bondon A., Baudy-Floc’H M., Felden B. (2019). Novel Antibiotics Effective Against Gram-positive and -negative Multi-resistant Bacteria with Limited Resistance. PLoS Biol..

[45] Chen C.H., Bepler T., Pepper K., Fu D., Lu T.K. (2022). Synthetic molecular evolution of antimicrobial peptides. Curr. Opin. Biotechnol..

[46] Shelburne C.E., An F.Y., Dhople V., Ramamoorthy A., Lopatin D.E., Lantz M.S. (2007). The spectrum of antimicrobial activity of bacteriocin subtilosin A. J. Antimicrob. Chemother..

[47] Mishra B., Lakshmaiah Narayana J., Lushnikova T., Wang X., Wang G. (2019). Low Cationicity Is Important for Systemic in Vivo Efficacy of Database-derived Peptides Against Drug-resistant Gram-positive Pathogens. Proc. Natl. Acad. Sci. USA.

[48] Schuster M., Brabet E., Oi K.K., Desjonquères N., Moehle K., Le Poupon K., Hell S., Gable S., Rithié V., Dillinger S. (2023). Peptidomimetic Antibiotics Disrupt the Lipopolysaccharide Transport Bridge of Drug-resistant Enterobacteriaceae. Sci. Adv..

[49] Dash R., Bhattacharjya S. (2021). Thanatin: An Emerging Host Defense Antimicrobial Peptide with Multiple Modes of Action. Lancet Infect. Dis..

[50] Nyembe P.L., Ntombela T., Makatini M.M. (2023). Review: Structure-activity Relationship of Antimicrobial Peptoids. Pharmaceutics.

[51] Spohn R., Daruka L., Lázár V., Martins A., Vidovics F., Grézal G., Méhi O., Kintses B., Számel M., Jangir P.K. (2019). Integrated Evolutionary Analysis Reveals Antimicrobial Peptides with Limited Resistance. Nat. Commun..

[52] Lázár V., Martins A., Spohn R., Daruka L., Grézal G., Fekete G., Számel M., Jangir P.K., Kintses B., Csörgő B. (2018). Antibiotic-resistant Bacteria Show Widespread Collateral Sensitivity to Antimicrobial Peptides. Nat. Microbiol..

[53] Gschwandtner M., Zhong S., Tschachler A., Mlitz V., Karner S., Elbe-Bürger A., Mildner M. (2014). Fetal Human Keratinocytes Produce Large Amounts of Antimicrobial Peptides: Involvement of Histone-methylation Processes. J. Investig. Dermatol..

[54] Underwood M., Bakaletz L. (2011). Innate Immunity and the Role of Defensins in Otitis Media. Curr. Allergy Asthma Rep..

[55] Jones D.E., Bevins C.L. (1993). Defensin-6 Mrna in Human Paneth Cells: Implications for Antimicrobia Peptides in Host Defense of the Human Bowel. FEBS Lett..

[56] Wang G., Li X., Wang Z. (2016). APD3: The Antimicrobial Peptide Database as a Tool for Research and Education. Nucleic Acids Res..

[57] Ibrahim H.R., Thomas U., Pellegrini A. (2001). A Helix-loop-helix Peptide at the Upper Lip of the Active Site Cleft of Lysozyme Confers Potent Antimicrobial Activity with Membrane Permeabilization Action. J. Biol. Chem..

[58] Pane K., Sgambati V., Zanfardino A., Smaldone G., Cafaro V., Angrisano T., Pedone E., Di Gaetano S., Capasso D., Haney E.F. (2016). A New Cryptic Cationic Antimicrobial Peptide from Human Apolipoprotein E with Antibacterial Activity and Immunomodulatory Effects on Human Cells. FEBS J..

[59] Sinha S., Harioudh M.K., Dewangan R.P., Ng W.J., Ghosh J.K., Bhattacharjya S. (2018). Cell-selective Pore Forming Antimicrobial Peptides of the Prodomain of Human Furin: A Conserved Aromatic/cationic Sequence Mapping, Membrane Disruption, and Atomic-resolution Structure and Dynamics. ACS Omega.

[60] Agerberth B., Gunne H., Odeberg J., Kogner P., Boman H.G., Gudmundsson G.H. (1995). FALL-39, a Putative Human Peptide Antibiotic, Is Cysteine-free and Expressed in Bone Marrow and Testis. Proc. Natl. Acad. Sci. USA.

[61] Larrick J.W., Hirata M., Balint R.F., Lee J., Zhong J., Wright S.C. (1995). Human CAP18: A Novel Antimicrobial Lipopolysaccharide-binding Protein. Infect. Immun..

[62] Cowland J.B., Johnsen A.H., Borregaard N. (1995). Hcap-18, a Cathelin/pro-bactenecin-like Protein of Human Neutrophil Specific Granules. FEBS Lett..

[63] Selsted M.E., Harwig S.S., Ganz T., Schilling J.W., Lehrer R.I. (1985). Primary Structures of Three Human Neutrophil Defensins. J. Clin. Investig..

[64] Wilde C.G., Griffith J.E., Marra M.N., Snable J.L., Scott R.W. (1989). Purification and characterization of human neutrophil peptide 4, a novel member of the defensin family. J. Biol. Chem..

[65] Jones D.E., Bevins C.L. (1992). Paneth cells of the human small intestine express an antimicrobial peptide gene. J. Biol. Chem..

[66] Oppenheim F.G., Xu T., McMillian F.M., Levitz S.M., Diamond R.D., Offner G.D., Troxler R.F. (1988). Histatins, a novel family of histidine-rich proteins in human parotid secretion. Isolation, characterization, primary structure, and fungistatic effects on *Candida albicans*. J. Biol. Chem..

[67] Bensch K.W., Raida M., Mägert H.-J., Schulz-Knappe P., Forssmann W.-G. (1995). Hbd-1: A Novel Β-defensin from Human Plasma. FEBS Lett..

[68] Harder J., Bartels J., Christophers E., Schröder J.-M. (1997). A Peptide Antibiotic from Human Skin. Nature.

[69] Harder J., Bartels J., Christophers E., Schröder J.-M. (2001). Isolation and Characterization of Human Μ-defensin-3, a Novel Human Inducible Peptide Antibiotic. J. Biol. Chem..

[70] García J.R., Krause A., Schulz S., Rodríguez-Jiménez F.J., Klüver E., Adermann K., Forssmann U., Frimpong-Boateng A., Bals R., Forssmann W.G. (2001). Human beta-defensin 4: A novel inducible peptide with a specific salt-sensitive spectrum of antimicrobial activity. FASEB J..

[71] Schittek B., Hipfel R., Sauer B., Bauer J., Kalbacher H., Stevanovic S., Schirle M., Schroeder K., Blin N., Meier F. (2001). Dermcidin: A Novel Human Antibiotic Peptide Secreted by Sweat Glands. Nat. Immunol..

[72] Stenger S., Hanson D.A., Teitelbaum R., Dewan P., Niazi K.R., Froelich C.J., Ganz T., Thoma-Uszynski S., Melián A., Bogdan C. (1998). An antimicrobial activity of cytolytic T cells mediated by granulysin. Science.

[73] Hieshima K., Ohtani H., Shibano M., Izawa D., Nakayama T., Kawasaki Y., Shiba F., Shiota M., Katou F., Saito T. (2003). CCL28 has dual roles in mucosal immunity as a chemokine with broad-spectrum antimicrobial activity. J. Immunol..

[74] Krijgsveld J., Zaat S.A.J., Meeldijk J., Van Veelen P.A., Fang G., Poolman B., Brandt E., Ehlert J.E., Kuijpers A.J., Engbers G.H.M. (2000). Thrombocidins, Microbicidal Proteins from Human Blood Platelets, Are C-terminal Deletion Products of CXC Chemokines. J. Biol. Chem..

[75] Krause A., Neitz S., Mägert H.-J., Schulz A., Forssmann W.-G., Schulz-Knappe P., Adermann K. (2000). LEAP-1, a Novel Highly Disulfide-bonded Human Peptide, Exhibits Antimicrobial Activity. FEBS Lett..

[76] Cutuli M., Cristiani S., Lipton J.M., Catania A. (2000). Antimicrobial Effects of A-msh Peptides. J. Leukoc. Biol..

[77] Lee E.Y., Chan L.C., Wang H., Lieng J., Hung M., Srinivasan Y., Wang J., Waschek J.A., Ferguson A.L., Lee K.-F. (2021). PACAP Is a Pathogen-inducible Resident Antimicrobial Neuropeptide Affording Rapid and Contextual Molecular Host Defense of the Brain. Proc. Natl. Acad. Sci. USA.

[78] Tam C., Mun J.J., Evans D.J., Fleiszig S.M.J. (2012). Cytokeratins Mediate Epithelial Innate Defense Through Their Antimicrobial Properties. J. Clin. Investig..

[79] Tollner T.L., Yudin A.I., Tarantal A.F., Treece C.A., Overstreet J.W., Cherr G.N. (2008). Beta-defensin 126 on the Surface of Macaque Sperm Mediates Attachment of Sperm to Oviductal Epithelia1. Biol. Reprod..

[80] Kościuczuk E.M., Lisowski P., Jarczak J., Strzałkowska N., Jóźwik A., Horbańczuk J., Krzyżewski J., Zwierzchowski L., Bagnicka E. (2012). Cathelicidins: Family of Antimicrobial Peptides. A Review. Mol. Biol. Rep..

[81] Zanetti M. (2004). Cathelicidins, multifunctional peptides of the innate immunity. J. Leukoc. Biol..

[82] Lenarčič B., Ritonja A., Dolenc I., Stoka V., Berbič S., Pungerčar J., Štrukelj B., Turk V. (1993). Pig Leukocyte Cysteine Proteinase Inhibitor (PLCPI), a New Member of the Stefin Family. FEBS Lett..

[83] Ritonja A., Kopitar M., Jerala R., Turk V. (1989). Primary Structure of a New Cysteine Proteinase Inhibitor from Pig Leucocytes. FEBS Lett..

[84] Storici P., Tossi A., Lenarčič B., Romeo D. (1996). Purification and Structural Characterization of Bovine Cathelicidins, Precursors of Antimicrobial Peptides. Eur. J. Biochem..

[85] Scocchi M., Wang S., Zanetti M. (1997). Structural Organization of the Bovine Cathelicidin Gene Family and Identification of a Novel Member1. FEBS Lett..

[86] Johansson J., Gudmundsson G.H., Rottenberg M.E., Berndt K.D., Agerberth B. (1998). Conformation-dependent Antibacterial Activity of the Naturally Occurring Human Peptide LL-37. J. Biol. Chem..

[87] Chen C., Brock R., Luh F., Chou P.-J., Larrick J.W., Huang R.-F., Huang T.-H. (1995). The Solution Structure of the Active Domain of CAP18—A Lipopolysaccharide Binding Protein from Rabbit Leukocytes. FEBS Lett..

[88] Mani R., Cady S.D., Tang M., Waring A.J., Lehrer R.I., Hong M. (2006). Membrane-dependent Oligomeric Structure and Pore Formation of a Β-hairpin Antimicrobial Peptide in Lipid Bilayers from Solid-state NMR. Proc. Natl. Acad. Sci. USA.

[89] Rozek A., Friedrich C.L., Hancock R.E. (2000). Structure of the bovine antimicrobial peptide indolicidin bound to dodecylphosphocholine and sodium dodecyl sulfate micelles. Biochemistry.

[90] Ramanathan B., Davis E.G., Ross C.R., Blecha F. (2002). Cathelicidins: Microbicidal activity, mechanisms of action, and roles in innate immunity. Microbes Infect..

[91] Xiao Y., Cai Y., Bommineni Y.R., Fernando S.C., Prakash O., Gilliland S.E., Zhang G. (2006). Identification and Functional Characterization of Three Chicken Cathelicidins with Potent Antimicrobial Activity. J. Biol. Chem..

[92] Bhunia A., Mohanram H., Bhattacharjya S. (2009). Lipopolysaccharide bound structures of the active fragments of fowlicidin-1, a cathelicidin family of antimicrobial and antiendotoxic peptide from chicken, determined by transferred nuclear Overhauser effect spectroscopy. Biopolymers.

[93] Bals R., Lang C., Weiner D.J., Vogelmeier C., Welsch U., Wilson J.M. (2001). Rhesus monkey (*Macaca mulatta*) mucosal antimicrobial peptides are close homologues of human molecules. Clin. Diagn. Lab. Immunol..

[94] Gallo R.L., Kim K.J., Bernfield M., Kozak C.A., Zanetti M., Merluzzi L., Gennaro R. (1997). Identification of CRAMP, a Cathelin-related Antimicrobial Peptide Expressed in the Embryonic and Adult Mouse. J. Biol. Chem..

[95] Nagaoka I., Tsutsumi-Ishii Y., Yomogida S., Yamashita T. (1997). Isolation of Cdna Encoding Guinea Pig Neutrophil Cationic Antibacterial Polypeptide of 11 Kda (CAP11) and Evaluation of CAP11 Mrna Expression During Neutrophil Maturation. J. Biol. Chem..

[96] Lawyer C., Pai S., Watabe M., Borgia P., Mashimo T., Eagleton L., Watabe K. (1996). Antimicrobial Activity of a 13 Amino Acid Tryptophan-rich Peptide Derived from a Putative Porcine Precursor Protein of a Novel Family of Antibacterial Peptides. FEBS Lett..

[97] Gidalevitz D., Ishitsuka Y., Muresan A.S., Konovalov O., Waring A.J., Lehrer R.I., Lee K.Y.C. (2003). Interaction of Antimicrobial Peptide Protegrin with Biomembranes. Proc. Natl. Acad. Sci. USA.

[98] Tossi A., Scocchi M., Zanetti M., Storici P., Gennaro R. (1995). PMAP-37, a Novel Antibacterial Peptide from Pig Myeloid Cells. Cdna Cloning, Chemical Synthesis and Activity. Eur. J. Biochem..

[99] Agerberth B., Lee J., Bergman T., Carlquist M., Boman H.G., Mutt V., Jörnvall H. (1991). Amino Acid Sequence of PR-39. Eur. J. Biochem..

[100] Romeo D., Skerlavaj B., Bolognesi M., Gennaro R. (1988). Structure and bactericidal activity of an antibiotic dodecapeptide purified from bovine neutrophils. J. Biol. Chem..

[101] Bagella L., Scocchi M., Zanetti M. (1995). Cdna Sequences of Three Sheep Myeloid Cathelicidins. FEBS Lett..

[102] Skerlavaj B., Gennaro R., Bagella L., Merluzzi L., Risso A., Zanetti M. (1996). Biological Characterization of Two Novel Cathelicidin-derived Peptides and Identification of Structural Requirements for Their Antimicrobial and Cell Lytic Activities. J. Biol. Chem..

[103] Thennarasu S., Tan A., Penumatchu R., Shelburne C.E., Heyl D.L., Ramamoorthy A. (2010). Antimicrobial and membrane disrupting activities of a peptide derived from the human cathelicidin antimicrobial peptide LL-37. Biophys. J..

[104] Travis S.M., Anderson N.N., Forsyth W.R., Espiritu C., Conway B.D., Greenberg E.P., Mccray P.B., Lehrer R.I., Welsh M.J., Tack B.F. (2000). Bactericidal Activity of Mammalian Cathelicidin-derived Peptides. Infect. Immun..

[105] Schlusselhuber M., Torelli R., Martini C., Leippe M., Cattoir V., Leclercq R., Laugier C., Grötzinger J., Sanguinetti M., Cauchard J. (2013). The Equine Antimicrobial Peptide Ecath1 Is Effective Against the Facultative Intracellular Pathogen Rhodococcus Equi in Mice. Antimicrob. Agents Chemother..

[106] Uzzell T., Stolzenberg E.D., Shinnar A.E., Zasloff M. (2003). Hagfish intestinal antimicrobial peptides are ancient cathelicidins. Peptides.

[107] Santana F.L., Estrada K., Alford M.A., Wu B.C., Dostert M., Pedraz L., Akhoundsadegh N., Kalsi P., Haney E.F., Straus S.K. (2022). Novel Alligator Cathelicidin As-cath8 Demonstrates Anti-infective Activity Against Clinically Relevant and Crocodylian Bacterial Pathogens. Antibiotics.

[108] Dürr U.H., Sudheendra U.S., Ramamoorthy A. (2006). LL-37, the only human member of the cathelicidin family of antimicrobial peptides. Biochim. Biophys. Acta Biomembr..

[109] Porcelli F., Verardi R., Shi L., Henzler-Wildman K.A., Ramamoorthy A., Veglia G. (2008). NMR Structure of the Cathelicidin-derived Human Antimicrobial Peptide LL-37 in Dodecylphosphocholine Micelles. Biochemistry.

[110] Ding B., Soblosky L., Nguyen K., Geng J., Yu X., Ramamoorthy A., Chen Z. (2013). Physiologically-relevant Modes of Membrane Interactions by the Human Antimicrobial Peptide, LL-37, Revealed by SFG Experiments. Sci. Rep..

[111] Wang G. (2008). Structures of Human Host Defense Cathelicidin LL-37 and Its Smallest Antimicrobial Peptide KR-12 in Lipid Micelles. J. Biol. Chem..

[112] Valore E.V., Park C.H., Quayle A.J., Wiles K.R., Mccray P.B., Ganz T. (1998). Human Beta-defensin-1: An Antimicrobial Peptide of Urogenital Tissues. J. Clin. Investig..

[113] Dhople V., Krukemeyer A., Ramamoorthy A. (2006). The human beta-defensin-3, an antibacterial peptide with multiple biological functions. Biochim. Biophys. Acta.

[114] Lehrer R.I., Lichtenstein A.K., Ganz T. (1993). Defensins: Antimicrobial and cytotoxic peptides of mammalian cells. Annu. Rev. Immunol..

[115] Gudmundsson G.H., Agerberth B., Odeberg J., Bergman T., Olsson B., Salcedo R. (1996). The Human Gene FALL39 and Processing of the Cathelin Precursor to the Antibacterial Peptide LL-37 in Granulocytes. Eur. J. Biochem..

[116] Sinha S., Zheng L., Mu Y., Ng W.J., Bhattacharjya S. (2017). Structure and Interactions of A Host Defense Antimicrobial Peptide Thanatin in Lipopolysaccharide Micelles Reveal Mechanism of Bacterial Cell Agglutination. Sci. Rep..

[117] Domadia P.N., Bhunia A., Ramamoorthy A., Bhattacharjya S. (2010). Structure, interactions, and antibacterial activities of MSI-594 derived mutant peptide MSI-594F5A in lipopolysaccharide micelles: Role of the helical hairpin conformation in outer-membrane permeabilization. J. Am. Chem. Soc..

[118] Bhunia A., Mohanram H., Domadia P.N., Torres J., Bhattacharjya S. (2009). Designed Β-boomerang Antiendotoxic and Antimicrobial Peptides. J. Biol. Chem..

[119] Ilyas H., Kim J., Lee D., Malmsten M., Bhunia A. (2019). Structural Insights into the Combinatorial Effects of Antimicrobial Peptides Reveal a Role of Aromatic–aromatic Interactions in Antibacterial Synergism. J. Biol. Chem..

[120] Datta A., Jaiswal N., Ilyas H., Debnath S., Biswas K., Kumar D., Bhunia A. (2017). Glycine-Mediated Short Analogue of a Designed Peptide in Lipopolysaccharide Micelles: Correlation Between Compact Structure and Anti-Endotoxin Activity. Biochemistry.

[121] Jakubec M., Rylandsholm F.G., Rainsford P., Silk M., Bril’Kov M., Kristoffersen T., Juskewitz E., Ericson J.U., Svendsen J.S.M. (2023). Goldilocks Dilemma: LPS Works Both as the Initial Target and a Barrier for the Antimicrobial Action of Cationic Amps on E. Coli. Biomolecules.

[122] Mares J., Kumaran S., Gobbo M., Zerbe O. (2009). Interactions of Lipopolysaccharide and Polymyxin Studied by NMR Spectroscopy. J. Biol. Chem..

[123] Swarbrick J.D., Karas J.A., Li J., Velkov T. (2020). Structure of Micelle Bound Cationic Peptides by NMR Spectroscopy Using a Lanthanide Shift Reagent. Chem. Commun..

[124] Yu K., Park K., Kang S.W., Shin S.Y., Hahm K.S., Kim Y. (2002). Solution structure of a cathelicidin-derived antimicrobial peptide, CRAMP as determined by NMR spectroscopy. J. Pept. Res..

[125] Park K., Oh D., Shin S.Y., Hahm K.S., Kim Y. (2002). Structural studies of porcine myeloid antibacterial peptide PMAP-23 and its analogues in DPC micelles by NMR spectroscopy. Biochem. Biophys. Res. Commun..

[126] Tack B.F., Sawai M.V., Kearney W.R., Robertson A.D., Sherman M.A., Wang W., Hong T., Boo L.M., Wu H., Waring A.J. (2002). SMAP-29 Has Two Lps-binding Sites and a Central Hinge. Eur. J. Biochem..

[127] Yang S., Lee C.W., Kim H.J., Jung H.H., Kim J.I., Shin S.Y., Shin S.H. (2019). Structural analysis and mode of action of BMAP-27, a cathelicidin-derived antimicrobial peptide. Peptides.

[128] Xiao Y., Dai H., Bommineni Y.R., Soulages J.L., Gong Y., Prakash O., Zhang G. (2006). Structure–activity Relationships of Fowlicidin-1, a Cathelicidin Antimicrobial Peptide in Chicken. FEBS J..

[129] Bommineni Y.R., Dai H., Gong Y.X., Soulages J.L., Fernando S.C., Desilva U., Prakash O., Zhang G. (2007). Fowlicidin-3 is an alpha-helical cationic host defense peptide with potent antibacterial and lipopolysaccharide-neutralizing activities. FEBS J..

[130] Saravanan R., Bhattacharjya S. (2011). Oligomeric structure of a cathelicidin antimicrobial peptide in dodecylphosphocholine micelle determined by NMR spectroscopy. Biochim. Biophys. Acta Biomembr..

[131] Chen J., Falla T.J., Liu H., Hurst M.A., Fujii C.A., Mosca D.A., Embree J.R., Loury D.J., Radel P.A., Cheng Chang C. (2000). Development of protegrins for the treatment and prevention of oral mucositis: Structure-activity relationships of synthetic protegrin analogues. Biopolymers.

[132] Hou M., Zhang N., Yang J., Meng X., Yang R., Li J., Sun T. (2013). Antimicrobial Peptide LL-37 and IDR-1 Ameliorate MRSA Pneumonia in Vivo. Cell. Physiol. Biochem..

[133] Aronen M., Viikari L., Langen H., Kohonen I., Wuorela M., Vuorinen T., Söderlund-Venermo M., Viitanen M., Camargo C.A., Vahlberg T. (2022). The Long-term Prognostic Value of Serum 25(OH)D, Albumin, and LL-37 Levels in Acute Respiratory Diseases Among Older Adults. BMC Geriatr..

[134] Zhu C., Zhou Y., Zhu J., Liu Y., Sun M. (2019). Proteína 3 Contendo Um Domínio NACHT, Porção C-terminal Rica Em Repetições De Leucina E De Domínio Pirina E LL-37: Valor Prognóstico De Novos Biomarcadores Em Pneumonia Adquirida Na Comunidade. J. Bras. Pneumol..

[135] Majewski K., Żelechowska P., Brzezińska-Błaszczyk E. (2017). Circulating Cathelicidin LL-37 in Adult Patients with Pulmonary Infectious Diseases. Clin. Investig. Med..

[136] Kozłowska E., Wysokiński A., Majewski K., Agier J., Margulska A., Brzezińska-Błaszczyk E. (2018). Human Cathelicidin LL-37—Does It Influence the Homeostatic Imbalance in Mental Disorders?. J. Biosci..

[137] Majewski K., Kozłowska E., Żelechowska P., Brzezińska-Błaszczyk E. (2018). Serum Concentrations of Antimicrobial Peptide Cathelicidin LL-37 in Patients with Bacterial Lung Infections. Cent. Eur. J. Immunol..

[138] Krasnodembskaya A., Song Y., Fang X., Gupta N., Serikov V., Lee J.-W., Matthay M.A. (2010). Antibacterial Effect of Human Mesenchymal Stem Cells Is Mediated in Part from Secretion of the Antimicrobial Peptide LL-37. Stem Cells.

[139] Mücke P.-A., Maaß S., Kohler T.P., Hammerschmidt S., Becher D. (2020). Proteomic Adaptation of Streptococcus Pneumoniae to the Human Antimicrobial Peptide LL-37. Microorganisms.

[140] Pouwels S.D., Hesse L., Wu X., Allam V.S.R.R., Van Oldeniel D., Bhiekharie L.J., Phipps S., Oliver B.G., Gosens R., Sukkar M.B. (2021). LL-37 and HMGB1 Induce Alveolar Damage and Reduce Lung Tissue Regeneration via RAGE. Am. J. Physiol. -Lung Cell. Mol. Physiol..

[141] Tatsuta M., Kan-o K., Ishii Y., Yamamoto N., Ogawa T., Fukuyama S., Ogawa A., Fujita A., Nakanishi Y., Matsumoto K. (2019). Effects of Cigarette Smoke on Barrier Function and Tight Junction Proteins in the Bronchial Epithelium: Protective Role of Cathelicidin LL-37. Respir. Res..

[142] Uysal P., Simsek G., Durmus S., Sozer V., Aksan H., Yurt S., Cuhadaroglu C., Kosar F., Gelisgen R., Uzun H. (2019). evaluation of Plasma Antimicrobial Peptide LL-37 and Nuclear Factor-kappab Levels in Stable Chronic Obstructive Pulmonary Disease. Int. J. Chronic Obstr. Pulm. Dis..

[143] Jiang Y.-Y., Xiao W., Zhu M.-X., Yang Z.-H., Pan X.-J., Zhang Y., Sun C.-C., Xing Y. (2012). The Effect of Human Antibacterial Peptide LL-37 in the Pathogenesis of Chronic Obstructive Pulmonary Disease. Respir. Med..

[144] Sun C., Zhu M., Yang Z., Pan X., Zhang Y., Wang Q., Xiao W. (2014). LL-37 Secreted by Epithelium Promotes Fibroblast Collagen Production: A Potential Mechanism of Small Airway Remodeling in Chronic Obstructive Pulmonary Disease. Lab. Investig..

[145] Li X., Huang X., Li L., Wu J., Yi W., Lai Y., Qin L. (2022). Ll-37-coupled Porous Composite Scaffold for the Treatment of Infected Segmental Bone Defect. Pharmaceutics.

[146] Barlow P.G., Svoboda P., Mackellar A., Nash A.A., York I.A., Pohl J., Davidson D.J., Donis R.O. (2011). Antiviral Activity and Increased Host Defense Against Influenza Infection Elicited by the Human Cathelicidin LL-37. PLoS ONE.

[147] Tripathi S., Tecle T., Verma A., Crouch E., White M., Hartshorn K.L. (2013). The Human Cathelicidin LL-37 Inhibits Influenza A Viruses Through a Mechanism Distinct from That of Surfactant Protein D or Defensins. J. Gen. Virol..

[148] White M.R., Tripathi S., Verma A., Kingma P., Takahashi K., Jensenius J., Thiel S., Wang G., Crouch E.C., Hartshorn K.L. (2017). Collectins, H-ficolin and LL-37 Reduce Influence Viral Replication in Human Monocytes and Modulate Virus-induced Cytokine Production. Innate Immun..

[149] Lee I.H., Jung Y.-J., Cho Y.G., Nou I.S., Huq M.A., Nogoy F.M., Kang K.-K. (2017). SP-LL-37, Human Antimicrobial Peptide, Enhances Disease Resistance in Transgenic Rice. PLoS ONE.

[150] Palusinska-Szysz M., Jurak M., Gisch N., Waldow F., Zehethofer N., Nehls C., Schwudke D., Koper P., Mazur A. (2022). The human LL-37 peptide exerts antimicrobial activity against Legionella micdadei interacting with membrane phospholipids. Biochim. Biophys. Acta Mol. Cell. Biol. Lipids.

[151] Tripathi S., Wang G., White M., Rynkiewicz M., Seaton B., Hartshorn K. (2015). Identifying the Critical Domain of LL-37 Involved in Mediating Neutrophil Activation in the Presence of Influenza Virus: Functional and Structural Analysis. PLoS ONE.

[152] Hu L.I., Stohl E.A., Seifert H.S. (2022). The Neisseria Gonorrhoeae Type IV Pilus Promotes Resistance to Hydrogen Peroxide- and Ll-37-mediated Killing by Modulating the Availability of Intracellular, Labile Iron. PLoS Pathog..

[153] Kiattiburut W., Zhi R., Lee S.G., Foo A.C., Hickling D.R., Keillor J.W., Goto N.K., Li W., Conlan W., Angel J.B. (2018). Antimicrobial Peptide LL-37 and Its Truncated Forms, GI-20 and GF-17, Exert Spermicidal Effects and Microbicidal Activity Against Neisseria Gonorrhoeae. Hum. Reprod..

[154] Pashapour A., Sardari S., Ehsani P. (2022). In Silicodesign and in Vitro Evaluation of Some Novel Amps Derived from Human LL-37 as Potential Antimicrobial Agents for Keratitis. Iran. J. Pharm. Res..

[155] Sharma P., Sharma N., Mishra P., Joseph J., Mishra D.K., Garg P., Roy S. (2019). Differential Expression of Antimicrobial Peptides in Streptococcus Pneumoniae Keratitis and Stat3-dependent Expression of LL-37 by Streptococcus Pneumoniae in Human Corneal Epithelial Cells. Pathogens.

[156] Oliveira P.N., Courrol D.S., Chura-Chambi R.M., Morganti L., Souza G.O., Franzolin M.R., Wunder E.A., Heinemann M.B., Barbosa A.S. (2021). Inactivation of the antimicrobial peptide LL-37 by pathogenic leptospira. Microb. Pathog..

[157] Moreno-Angarita A., Aragón C.C., Tobón G.J. (2020). Cathelicidin ll-37: A new important molecule in the pathophysiology of systemic lupus erythematosus. J. Transl. Autoimmun..

[158] Kahlenberg J.M., Kaplan M.J. (2013). Little Peptide, Big Effects: The Role of LL-37 in Inflammation and Autoimmune Disease. J. Immunol..

[159] Lande R., Ganguly D., Facchinetti V., Frasca L., Conrad C., Gregorio J., Meller S., Chamilos G., Sebasigari R., Riccieri V. (2011). Neutrophils Activate Plasmacytoid Dendritic Cells by Releasing Self-dna–peptide Complexes in Systemic Lupus Erythematosus. Sci. Transl. Med..

[160] Pahar B., Madonna S., Das A., Albanesi C., Girolomoni G. (2020). Immunomodulatory Role of the Antimicrobial LL-37 Peptide in Autoimmune Diseases and Viral Infections. Vaccines.

[161] Jones A., Geörg M., Maudsdotter L., Jonsson A.-B. (2009). Endotoxin, Capsule, and Bacterial Attachment Contribute to Neisseria Meningitidis Resistance to the Human Antimicrobial Peptide LL-37. J. Bacteriol..

[162] Zughaier S.M., Svoboda P., Pohl J., Stephens D.S., Shafer W.M. (2010). The Human Host Defense Peptide LL-37 Interacts with Neisseria Meningitidis Capsular Polysaccharides and Inhibits Inflammatory Mediators Release. PLoS ONE.

[163] Seib K.L., Serruto D., Oriente F., Delany I., Adu-Bobie J., Veggi D., Aricò B., Rappuoli R., Pizza M. (2009). Factor H-binding Protein Is Important for Meningococcal Survival in Human Whole Blood and Serum and in the Presence of the Antimicrobial Peptide LL-37. Infect. Immun..

[164] Gutner M., Chaushu S., Balter D., Bachrach G. (2009). Saliva Enables the Antimicrobial Activity of LL-37 in the Presence of Proteases of Porphyromonas Gingivalis. Infect. Immun..

[165] Chinipardaz Z., Zhong J.M., Yang S. (2022). Regulation of LL-37 in Bone and Periodontium Regeneration. Life.

[166] Tada H., Shimizu T., Matsushita K., Takada H. (2017). Porphyromonas Gingivalisinduced IL-33 Down-regulates Hcap-18/ll-37 Production in Human Gingival Epithelial cells. Biomed. Res..

[167] Bedran T.B.L., Mayer M.P.A., Spolidorio D.P., Grenier D. (2014). Synergistic Anti-inflammatory Activity of the Antimicrobial Peptides Human Beta-defensin-3 (hbd-3) and Cathelicidin (LL-37) in a Three-dimensional Co-culture Model of Gingival Epithelial Cells and Fibroblasts. PLoS ONE.

[168] Puklo M., Guentsch A., Hiemstra P.S., Eick S., Potempa J. (2008). Analysis of Neutrophil-derived Antimicrobial Peptides in Gingival Crevicular Fluid Suggests Importance of Cathelicidin LL-37 in the Innate Immune Response Against Periodontogenic Bacteria. Oral Microbiol. Immunol..

[169] Lao J., Xie Z., Qin Q., Qin R., Li S., Yuan Y. (2023). Serum LL-37 and Inflammatory Cytokines Levels in Psoriasis. Immun. Inflamm. Dis..

[170] Dombrowski Y., Schauber J. (2012). Cathelicidin LL-37: A Defense Molecule with a Potential Role in Psoriasis Pathogenesis. Exp. Dermatol..

[171] Morizane S., Yamasaki K., Mühleisen B., Kotol P.F., Murakami M., Aoyama Y., Iwatsuki K., Hata T., Gallo R.L. (2012). Cathelicidin Antimicrobial Peptide LL-37 in Psoriasis Enables Keratinocyte Reactivity Against TLR9 Ligands. J. Investig. Dermatol..

[172] Hoffmann M.H., Bruns H., Bäckdahl L., Neregård P., Niederreiter B., Herrmann M., Catrina A.I., Agerberth B., Holmdahl R. (2013). The Cathelicidins LL-37 and Rcramp Are Associated with Pathogenic Events of Arthritis in Humans and Rats. Ann. Rheum. Dis..

[173] Cheah C.W., Al-Maleki A.R., Vaithilingam R.D., Vadivelu J., Sockalingam S., Baharuddin N.A., Bartold P.M. (2022). Associations Between Inflammation-related LL-37 with Subgingival Microbial Dysbiosis in Rheumatoid Arthritis Patients. Clin. Oral Investig..

[174] Chow L.N.Y., Choi K.-Y., Piyadasa H., Bossert M., Uzonna J., Klonisch T., Mookherjee N. (2014). Human Cathelicidin Ll-37-derived Peptide IG-19 Confers Protection in a Murine Model of Collagen-induced Arthritis. Mol. Immunol..

[175] Kuensaen C., Chomdej S., Kongdang P., Sirikaew N., Jaitham R., Thonghoi S., Ongchai S. (2019). LL-37 Alone and in Combination with IL17A Enhances Proinflammatory Cytokine Expression in Parallel with Hyaluronan Metabolism in Human Synovial Sarcoma Cell Line SW982—A Step Toward Understanding the Development of Inflammatory Arthritis. PLoS ONE.

[176] Koziel J., Bryzek D., Sroka A., Maresz K., Glowczyk I., Bielecka E., Kantyka T., Pyrć K., Svoboda P., Pohl J. (2014). Citrullination Alters Immunomodulatory Function of LL-37 Essential for Prevention of Endotoxin-Induced Sepsis. J. Immunol..

[177] Leite M.L., Duque H.M., Rodrigues G.R., da Cunha N.B., Franco O.L. (2023). The LL-37 domain: A clue to cathelicidin immunomodulatory response?. Peptide.

[178] Hu Z., Murakami T., Suzuki K., Tamura H., Kuwahara-Arai K., Iba T., Nagaoka I. (2014). Antimicrobial Cathelicidin Peptide LL-37 Inhibits the Lps/atp-induced Pyroptosis of Macrophages by Dual Mechanism. PLoS ONE.

[179] Nagaoka I., Tamura H., Reich J. (2020). Therapeutic Potential of Cathelicidin Peptide LL-37, an Antimicrobial Agent, in a Murine Sepsis Model. Lancet Infect. Dis..

[180] Rivas-Santiago B., Hernandez-Pando R., Carranza C., Juarez E., Contreras J.L., Aguilar-Leon D., Torres M., Sada E. (2008). Expression of Cathelicidin LL-37 Duringmycobacterium Tuberculosisinfection in Human Alveolar Macrophages, Monocytes, Neutrophils, and Epithelial Cells. Infect. Immun..

[181] Rekha R.S., Rao Muvva S.J., Wan M., Raqib R., Bergman P., Brighenti S., Gudmundsson G.H., Agerberth B. (2015). Phenylbutyrate Induces Ll-37-dependent Autophagy and Intracellular Killing of Mycobacterium Tuberculosis in Human Macrophages. Autophagy.

[182] Torres-Juarez F., Cardenas-Vargas A., Montoya-Rosales A., González-Curiel I., Garcia-Hernandez M.H., Enciso-Moreno J.A., Hancock R.E.W., Rivas-Santiago B. (2015). LL-37 Immunomodulatory Activity During Mycobacterium Tuberculosis Infection in Macrophages. Infect. Immun..

[183] Dhiman A., Talukdar S., Chaubey G.K., Dilawari R., Modanwal R., Chaudhary S., Patidar A., Boradia V.M., Kumbhar P., Raje C.I. (2023). Regulation of Macrophage Cell Surface GAPDH Alters LL-37 Internalization and Downstream Effects in the Cell. J. Innate Immun..

[184] Duan Z., Fang Y., Sun Y., Luan N., Chen X., Chen M., Han Y., Yin Y., Mwangi J., Niu J. (2018). Antimicrobial peptide LL-37 forms complex with bacterial DNA to facilitate blood translocation of bacterial DNA and aggravate ulcerative colitis. Sci. Bull..

[185] Memariani H., Memariani M. (2023). Antibiofilm Properties of Cathelicidin LL-37: An In-depth Review. World J. Microbiol. Biotechnol..

[186] Overhage J., Campisano A., Bains M., Torfs E.C.W., Rehm B.H.A., Hancock R.E.W. (2008). Human Host Defense Peptide LL-37 Prevents Bacterial Biofilm Formation. Infect. Immun..

[187] Schmidtchen A., Frick I., Andersson E., Tapper H., Björck L. (2002). Proteinases of Common Pathogenic Bacteria Degrade and Inactivate the Antibacterial Peptide LL-37. Mol. Microbiol..

[188] Sieprawska-Lupa M., Mydel P., Krawczyk K., Wójcik K., Puklo M., Lupa B., Suder P., Silberring J., Reed M., Pohl J. (2004). Degradation of Human Antimicrobial Peptide LL-37 by *Staphylococcus Aureus* -derived Proteinases. Antimicrob. Agents Chemother..

[189] Koziel J., Karim A.Y., Przybyszewska K., Ksiazek M., Rapala-Kozik M., Nguyen K.-A., Potempa J. (2010). Proteolytic Inactivation of LL-37 by Karilysin, a Novel Virulence Mechanism of *Tannerella forsythia*. J. Innate Immun..

[190] Thomassin J.-L., Brannon J.R., Gibbs B.F., Gruenheid S., Le Moual H. (2012). Ompt Outer Membrane Proteases of Enterohemorrhagic and Enteropathogenic Escherichia Coli Contribute Differently to the Degradation of Human LL-37. Infect. Immun..

[191] Brannon J.R., Thomassin J.-L., Desloges I., Gruenheid S., Le Moual H. (2013). Role of Uropathogenicescherichia Coliompt in the Resistance Against Human Cathelicidin LL-37. FEMS Microbiol. Lett..

[192] Papo N., Shahar M., Eisenbach L., Shai Y. (2003). A Novel Lytic Peptide Composed of Dl-amino Acids Selectively Kills Cancer Cells in Culture and in Mice. J. Biol. Chem..

[193] Makovitzki A., Fink A., Shai Y. (2009). Suppression of Human Solid Tumor Growth in Mice by Intratumor and Systemic Inoculation of Histidine-rich and Ph-dependent Host Defense–like Lytic Peptides. Cancer Res..

[194] Kamarajan P., Hayami T., Matte B., Liu Y., Danciu T., Ramamoorthy A., Worden F., Kapila S., Kapila Y. (2015). Nisin ZP, a Bacteriocin and Food Preservative, Inhibits Head and Neck Cancer Tumorigenesis and Prolongs Survival. PLoS ONE.

[195] Wang G., Vaisman I.I., Van Hoek M.L. (2022). Machine Learning Prediction of Antimicrobial Peptides. Single Cell Analysis.

[196] Heilborn J.D., Nilsson M.F., Jimenez C.I.C., Sandstedt B., Borregaard N., Tham E., Sørensen O.E., Weber G., Ståhle M. (2005). Antimicrobial Protein Hcap18/ll-37 Is Highly Expressed in Breast Cancer and Is a Putative Growth Factor for Epithelial Cells. Int. J. Cancer.

[197] Chen X., Zou X., Qi G., Tang Y., Guo Y., Si J., Liang L. (2018). Roles and Mechanisms of Human Cathelicidin LL-37 in Cancer. Cell. Physiol. Biochem..

[198] Piktel E., Niemirowicz K., Wnorowska U., Wątek M., Wollny T., Głuszek K., Góźdź S., Levental I., Bucki R. (2016). The Role of Cathelicidin LL-37 in Cancer Development. Arch. Immunol. Ther. Exp..

[199] Wu W.K.K., Wang G., Coffelt S.B., Betancourt A.M., Lee C.W., Fan D., Wu K., Yu J., Sung J.J.Y., Cho C.H. (2010). Emerging Roles of the Host Defense Peptide LL-37 in Human Cancer and Its Potential Therapeutic Applications. Int. J. Cancer.

[200] Verjans E.T., Zels S., Luyten W., Landuyt B., Schoofs L. (2016). Molecular mechanisms of LL-37-induced receptor activation: An overview. Peptides.

[201] Doss M., White M.R., Tecle T., Hartshorn K.L. (2009). Human Defensins and LL-37 in Mucosal Immunity. J. Leukoc. Biol..

[202] Wang G., Narayana J.L., Mishra B., Zhang Y., Wang F., Wang C., Zarena D., Lushnikova T., Wang X. (2019). Design of Antimicrobial Peptides: Progress Made with Human Cathelicidin LL-37. Advances in Experimental Medicine and Biology.

[203] Yang B., Good D., Mosaiab T., Liu W., Ni G., Kaur J., Liu X., Jessop C., Yang L., Fadhil R. (2020). Significance of LL-37 on Immunomodulation and Disease Outcome. BioMed Res. Int..

[204] Bucki R., Leszczyńska K., Namiot A., Sokołowski W. (2010). Cathelicidin LL-37: A Multitask Antimicrobial Peptide. Arch. Immunol. Ther. Exp..

[205] De Lorenzi E., Chiari M., Colombo R., Cretich M., Sola L., Vanna R., Gagni P., Bisceglia F., Morasso C., Lin J.S. (2017). Evidence That the Human Innate Immune Peptide LL-37 May Be a Binding Partner of Amyloid-β and Inhibitor of Fibril Assembly. J. Alzheimer’s Dis..

[206] Chen X., Deng S., Wang W., Castiglione S., Duan Z., Luo L., Cianci F., Zhang X., Xu J., Li H. (2022). Human Antimicrobial Peptide LL-37 Contributes to Alzheimer’s Disease Progression. Mol. Psychiatry.

[207] Armiento V., Hille K., Naltsas D., Lin J.S., Barron A.E., Kapurniotu A. (2020). The Human Host-defense Peptide Cathelicidin LL-37 Is a Nanomolar Inhibitor of Amyloid Self-assembly of Islet Amyloid Polypeptide (IAPP). Angew. Chem. Int. Ed..

[208] Sancho-Vaello E., François P., Bonetti E.-J., Lilie H., Finger S., Gil-Ortiz F., Gil-Carton D., Zeth K. (2017). Structural Remodeling and Oligomerization of Human Cathelicidin on Membranes Suggest Fibril-like Structures as Active Species. Sci. Rep..

[209] Sancho-Vaello E., Gil-Carton D., François P., Bonetti E.-J., Kreir M., Pothula K.R., Kleinekathöfer U., Zeth K. (2020). The Structure of the Antimicrobial Human Cathelicidin LL-37 Shows Oligomerization and Channel Formation in the Presence of Membrane Mimics. Sci. Rep..

[210] Li X., Li Y., Han H., Miller D.W., Wang G. (2006). Solution Structures of Human LL-37 Fragments and NMR-Based Identification of a Minimal Membrane-Targeting Antimicrobial and Anticancer Region. J. Am. Chem. Soc..

[211] Gunasekera S., Muhammad T., Strömstedt A.A., Rosengren K.J., Göransson U. (2020). Backbone Cyclization and Dimerization of Ll-37-derived Peptides Enhance Antimicrobial Activity and Proteolytic Stability. Front. Microbiol..

[212] Henzler Wildman K.A., Lee D.K., Ramamoorthy A. (2003). Mechanism of bilayer disruption by the human antimicrobial peptide, LL-37. Biochemistry.

[213] Nielsen J.E., Alford M.A., Yung D.B.Y., Molchanova N., Fortkort J.A., Lin J.S., Diamond G., Hancock R.E.W., Jenssen H., Pletzer D. (2022). Self-assembly of Antimicrobial Peptoids Impacts Their Biological Effects on ESKAPE Bacterial Pathogens. ACS Infect. Dis..

[214] Jiang X., Yang C., Qiu J., Ma D., Xu C., Hu S., Han W., Yuan B., Lu Y. (2022). Nanomolar LL-37 Induces Permeability of a Biomimetic Mitochondrial Membrane. Nanoscale.

[215] Mitra A., Paul S. (2023). Pathways of hLL-37_17-29_ Aggregation Give Insight into the Mechanism of α-Amyloid Formation. J. Phys. Chem. B.

[216] Li X., Li Y., Peterkosfsky A., Wang G. (2006). NMR studies of aurein 1.2 analogs. Biochim. Biophys. Acta Biomembr..

[217] Engelberg Y., Landau M. (2020). The Human LL-37(17-29) Antimicrobial Peptide Reveals a Functional Supramolecular Structure. Nat. Commun..

[218] Engelberg Y., Ragonis-Bachar P., Landau M. (2022). Rare by Natural Selection: Disulfide-bonded Supramolecular Antimicrobial Peptides. Biomacromolecules.

[219] Yun H., Min H.J., Lee C.W. (2020). NMR Structure and Bactericidal Activity of KR-12 Analog Derived from Human LL-37 as a Potential Cosmetic Preservative. J. Anal. Sci. Technol..

[220] Shcherbakov A.A., Spreacker P.J., Dregni A.J., Henzler-Wildman K.A., Hong M. (2022). High-ph Structure of Emre Reveals the Mechanism of Proton-coupled Substrate Transport. Nat. Commun..

[221] Nishiyama Y., Hou G., Agarwal V., Su Y., Ramamoorthy A. (2023). Ultrafast Magic Angle Spinning Solid-State NMR Spectroscopy: Advances in Methodology and Applications. Chem. Rev..

[222] Hellmich U.A., Lyubenova S., Kaltenborn E., Doshi R., van Veen H.W., Prisner T.F., Glaubitz C. (2013). Probing the ATP Hydrolysis Cycle of the ABC Multidrug Transporter LmrA by Pulsed EPR Spectroscopy. J. Am. Chem. Soc..

[223] Rogawski R., MecDermott A.E. (2017). New NMR tools for protein structure and function: Spin tags for dynamic nuclear polarization solid state NMR. Arch. Biochem. Biophys..

[224] Reif B., Ashbrook S.E., Emsley L., Hong M. (2021). Solid-state NMR Spectroscopy. Nat. Rev. Methods Primers.

[225] Gopinath T., Weber D., Wang S., Larsen E., Veglia G. (2021). Solid-State NMR of Membrane Proteins in Lipid Bilayers: To Spin or Not to Spin?. Acc. Chem. Res..

[226] Roversi D., Troiano C., Salnikov E., Giordano L., Riccitelli F., De Zotti M., Casciaro B., Loffredo M.R., Park Y., Formaggio F. (2023). Effects of antimicrobial peptides on membrane dynamics: A comparison of fluorescence and NMR experiments. Biophys. Chem..

[227] Salnikov E., Aisenbrey C., Bechinger B. (2022). Lipid saturation and head group composition have a pronounced influence on the membrane insertion equilibrium of amphipathic helical polypeptides. Biochim. Biophys. Acta Biomembr..

[228] Schweigardt F., Strandberg E., Wadhwani P., Reichert J., Bürck J., Cravo H.L.P., Burger L., Ulrich A.S. (2022). Membranolytic Mechanism of Amphiphilic Antimicrobial Β-stranded [kl]n Peptides. Biomedicines.

[229] Ramamoorthy A. (2009). Beyond NMR Spectra of Antimicrobial Peptides: Dynamical Images at Atomic Resolution and Functional Insights. Solid State Nucl. Magn. Reson..

[230] Mihailescu M., Sorci M., Seckute J., Silin V.I., Hammer J., Perrin B.S., Hernandez J.I., Smajic N., Shrestha A., Bogardus K.A. (2019). Structure and Function in Antimicrobial Piscidins: Histidine Position, Directionality of Membrane Insertion, and Ph-dependent Permeabilization. J. Am. Chem. Soc..

[231] Xhindoli D., Morgera F., Zinth U., Rizzo R., Pacor S., Tossi A. (2015). New Aspects of the Structure and Mode of Action of the Human Cathelicidin LL-37 Revealed by the Intrinsic Probe P-cyanophenylalanine. Biochem. J..

[232] Oren Z., Lerman J.C., Gudmundsson G.H., Agerberth B., Shai Y. (1999). Structure and Organization of the Human Antimicrobial Peptide LL-37 in Phospholipid Membranes: Relevance to the Molecular Basis for Its Non-cell-selective Activity. Biochem. J..

[233] Sood R., Domanov Y., Pietiäinen M., Kontinen V.P., Kinnunen P.K. (2008). Binding of LL-37 to model biomembranes: Insight into target vs host cell recognition. Biochim. Biophys. Acta.

[234] Liu C., Henning-Knechtel A., Österlund N., Wu J., Wang G., Gräslund R.A.O., Kirmizialtin S., Luo J. (2023). Oligomer Dynamics of LL-37 Truncated Fragments Probed by A-hemolysin Pore and Molecular Simulations. Small.

[235] Zeth K., Sancho-Vaello E. (2017). The Human Antimicrobial Peptides Dermcidin and LL-37 Show Novel Distinct Pathways in Membrane Interactions. Front. Chem..

[236] Xhindoli D., Pacor S., Benincasa M., Scocchi M., Gennaro R., Tossi A. (2016). The human cathelicidin LL-37—A pore-forming antibacterial peptide and host-cell modulator. Biochim. Biophys. Acta.

[237] Henzler-Wildman K.A., Martinez G.V., Brown M.F., Ramamoorthy A. (2004). Perturbation of the hydrophobic core of lipid bilayers by the human antimicrobial peptide LL-37. Biochemistry.

[238] Hardy J., Selkoe D.J. (2002). The amyloid hypothesis of Alzheimer’s disease: Progress and problems on the road to therapeutics. Science.

[239] Michele, Samuel, Jeffrey, Chen J., Lee D.-K., Ramamoorthy A. (2012). Two-step Mechanism of Membrane Disruption by Aβ Through Membrane Fragmentation and Pore Formation. Biophys. J..

[240] Kotler S.A., Brender J.R., Vivekanandan S., Suzuki Y., Yamamoto K., Monette M., Krishnamoorthy J., Walsh P., Cauble M., Holl M.M.B. (2015). High-resolution NMR Characterization of Low Abundance Oligomers of Amyloid-β Without Purification. Sci. Rep..

[241] Colombo L., Gamba A., Cantù L., Salmona M., Tagliavini F., Rondelli V., Del Favero E., Brocca P. (2017). Pathogenic Aβ A2V Versus Protective Aβ A2T Mutation: Early Stage Aggregation and Membrane Interaction. Biophys. Chem..

[242] Ma L., Li X., Peterson R.B., Peng A., Huang K. (2023). Probing the interactions between amyloidogenic proteins and bio-membranes. Biophys. Chem..

[243] Fatafta H., Kav B., Bundschuh B.F., Loschwitz J., Strodel B. (2022). Disorder-to-order transition of the amyloid-β peptide upon lipid binding. Biophys. Chem..

[244] Zambrano P., Jemiola-Rzeminska M., Muñoz-Torrero D., Suwalsky M., Strzalka K. (2023). A rhein-huprine hybrid protects erythrocyte membrane integrity against Alzheimer’s disease related Aβ(1-42) peptide. Biophys. Chem..

[245] Nicastro M.C., Spigolon D., Librizzi F., Moran O., Ortore M.G., Bulone D., Biagio P.L., Carrotta R. (2016). Amyloid β-peptide insertion in liposomes containing GM1-cholesterol domains. Biophys. Chem..

[246] Saha J., Ford B.J., Wang X., Boyd S., Morgan S.E., Rangachari V. (2023). Sugar distributions on gangliosides guide the formation and stability of amyloid-β oligomers. Biophys. Chem..

[247] Kenyaga J.M., Oteino S.A., Sun Y., Qiang W. (2023). In-cell 31P solid-state NMR measurements of the lipid dynamics and influence of exogeneous β-amyloid peptides on live neuroblastoma neuro-2a cells. Biophys. Chem..

[248] Morita M., Vestergaard M., Hamada T., Takagi M. (2009). Real-time observation of model membrane dynamics induced by Alzheimer’s amyloid beta. Biophys. Chem..

[249] Kumar M., Ivanova M.I., Ramamoorthy A. (2023). Non-micellar ganglioside GM1 induces an instantaneous conformational change in Aβ42 leading to the modulation of the peptide amyloid-fibril pathway. Biophys. Chem..

[250] Kumar M., I Ivanova M., Ramamoorthy A. (2023). Ganglioside GM1 Produces Stable, Short, and Cytotoxic Aβ40 Protofibrils. Chem. Commun..

[251] Lee M., Shi X., Barron A.E., McGeer E., McGeer P.L. (2015). Human antimicrobial peptide LL-37 induces glial-mediated neuroinflammation. Biochem. Pharmacol..

[252] Fülöp T., Itzhaki R.F., Balin B.J., Miklossy J., Barron A.E. (2018). Role of Microbes in the Development of Alzheimer’s Disease: State of the Art—An International Symposium Presented at the 2017 IAGG Congress in San Francisco. Front. Genet..

[253] Betsholtz C., Johnson K.H., Westermark P. (1989). ‘amylin’ Hormone. Nature.

[254] Westermark G.T., Westermark P. (2013). Islet amyloid polypeptide and diabetes. Curr. Protein Pept. Sci..

[255] Westermark P., Andersson A., Westermark G.T. (2005). Is Aggregated IAPP a Cause of Beta-cell Failure in Transplanted Human Pancreatic Islets?. Curr. Diabetes Rep..

[256] Milardi D., Gazit E., Radford S.E., Xu Y., Gallardo R.U., Caflisch A., Westermark G.T., Westermark P., Rosa C.L., Ramamoorthy A. (2021). Proteostasis of Islet Amyloid Polypeptide: A Molecular Perspective of Risk Factors and Protective Strategies for Type II Diabetes. Chem. Rev..

[257] Pithadia A., Brender J.R., Fierke C.A., Ramamoorthy A. (2016). Inhibition of IAPP Aggregation and Toxicity by Natural Products and Derivatives. J. Diabetes Res..

[258] Sciacca M.F.M., Chillemi R., Sciuto S., Greco V., Messineo C., Kotler S.A., Lee D., Brender J.R., Ramamoorthy A., Rosa C.L. (2018). A blend of two resveratrol derivatives abolishes hIAPP amyloid growth and membrane damage. Biochim. Biophys. Acta Biomembr..

[259] Cox S.J., Rodriguez Camargo D.C., Lee Y.-H., Dubini R.C.A., Rovó P., Ivanova M.I., Padmini V., Reif B., Ramamoorthy A. (2020). Small Molecule Induced Toxic Human-iapp Species Characterized by NMR. Chem. Commun..

[260] Tsai H., Huang C., Tu L. (2024). TPE conjugated islet amyloid polypeptide probe for detection of peptide oligomers. Biophys. Chem..

[261] Yu F., Teng Y., Yang S., He Y., Zhang Z., Yang H., Ding C., Zhou P. (2022). The thermodynamic and kinetic mechanisms of a Ganoderma lucidum proteoglycan inhibiting hIAPP amyloidosis. Biophys. Chem..

[262] Nireeksha, Hegde M.N., Kumari N.S. (2024). Potential Role of Salivary Vitamin D Antimicrobial Peptide LL-37 and Interleukins in Severity of Dental Caries: An Exvivo Study. BMC Oral Health.

[263] Juszczak M., Zawrotniak M., Rapala-Kozik M. (2024). Complexation of Fungal Extracellular Nucleic Acids by Host LL-37 Peptide Shapes Neutrophil Response to Candida Albicans Biofilm. Front. Immunol..

[264] Song Y., Zhang S., Zhao N., Nong C., He Y., Bao R. (2024). Pseudomonas Aeruginosa Two-component System Cprrs Regulates Higba Expression and Bacterial Cytotoxicity in Response to LL-37 Stress. PLoS Pathog..

[265] Zhang Y., Bharathi V., Dokoshi T., De Anda J., Ursery L.T., Kulkarni N.N., Nakamura Y., Chen J., Luo E.W.C., Wang L. (2024). Viral Afterlife: SARS-CoV-2 as a Reservoir of Immunomimetic Peptides That Reassemble into Proinflammatory Supramolecular Complexes. Proc. Natl. Acad. Sci. USA.

[266] Lei R., Yang C., Sun Y., Li D., Hao L., Li Y., Wu S., Li H., Lan C., Fang X. (2024). Turning Cationic Antimicrobial Peptide KR-12 into Self-assembled Nanobiotics with Potent Bacterial Killing and LPS Neutralizing Activities. Nanoscale.

